# Evaluation of the Association of Platelet Count, Mean Platelet Volume, and Platelet Transfusion With Intraventricular Hemorrhage and Death Among Preterm Infants

**DOI:** 10.1001/jamanetworkopen.2022.37588

**Published:** 2022-10-19

**Authors:** Chong Chen, Sicheng Wu, Jia Chen, Jinghui Wu, Yabo Mei, Tao Han, Changshuan Yang, Xilin Ouyang, May Chun Mei Wong, Zhichun Feng

**Affiliations:** 1 Department of Neonatology, Faculty of Pediatrics, Seventh Medical Center of PLA General Hospital, Beijing, China; 2 National Engineering Laboratory for Birth Defects Prevention and Control of Key Technology, Beijing, China; 3 Beijing Key Laboratory of Pediatric Organ Failure, Beijing, China; 4 Dental Public Health, Faculty of Dentistry, the University of Hong Kong, Hong Kong Special Administrative Region, China; 5 Department of Blood Transfusion, Fourth Medical Center of PLA General Hospital, Beijing, China

## Abstract

**Importance:**

Platelet transfusion is commonly performed in infants to correct severe thrombocytopenia or prevent bleeding. Exploring the associations of platelet transfusion, platelet count (PC), and mean platelet volume (MPV) with intraventricular hemorrhage (IVH) and in-hospital mortality in preterm infants can provide evidence for the establishment of future practices.

**Objectives:**

To evaluate the associations of platelet transfusion, PC, and MPV with IVH and in-hospital mortality and to explore whether platelet transfusion–associated IVH and mortality risks vary with PC and MPV levels at the time of transfusion.

**Design, Setting, and Participants:**

This retrospective cohort study included preterm infants who were transferred to the neonatal intensive care unit on their day of birth and received ventilation during their hospital stay. The study was conducted at a neonatal intensive care unit referral center in Beijing, China, between May 2016 and October 2017. Data were retrieved and analyzed from December 2020 to January 2022.

**Exposures:**

Platelet transfusion, PC, and MPV.

**Main Outcomes and Measures:**

Any grade IVH, severe IVH (grade 3 or 4), and in-hospital mortality.

**Results:**

Among the 1221 preterm infants (731 [59.9%] male; median [IQR] gestational age, 31.0 [29.0-33.0] weeks), 94 (7.7%) received 166 platelet transfusions. After adjustment for potential confounders, platelet transfusion was significantly associated with mortality (hazard ratio [HR], 1.48; 95% CI, 1.13-1.93; *P* = .004). A decreased PC was significantly associated with any grade IVH (HR per 50 × 10^3^/μL, 1.13; 95% CI, 1.05-1.22; *P* = .001), severe IVH (HR per 50 × 10^3^/μL, 1.16; 95% CI, 1.02-1.32; *P* = .02), and mortality (HR per 50 × 10^3^/μL, 1.74; 95% CI, 1.48-2.03; *P* < .001). A higher MPV was associated with a lower risk of mortality (HR, 0.83; 95% CI, 0.69-0.98; *P* = .03). The platelet transfusion–associated risks for both IVH and mortality increased when transfusion was performed in infants with a higher PC level (eg, PC of 25 × 10^3^/μL: HR, 1.20; 95% CI, 0.89-1.62; PC of 100 × 10^3^/μL: HR, 1.40; 95% CI, 1.08-1.82). The platelet transfusion–associated risks of IVH and mortality varied with MPV level at the time of transfusion.

**Conclusions and Relevance:**

In preterm infants, platelet transfusion, PC, and MPV were associated with mortality, and PC was also associated with any grade IVH and severe IVH. The findings suggest that a lower platelet transfusion threshold is preferred; however, the risk of a decreased PC should not be ignored.

## Key Points

**Question:**

What are the associations of platelet transfusion, platelet count (PC), and mean platelet volume (MPV) with intraventricular hemorrhage (IVH) and mortality in preterm infants?

**Findings:**

In this cohort study of 1221 preterm infants who received ventilation, platelet transfusion was associated with an increased risk of mortality, while decreased PC was associated with increased risks of IVH and mortality. The platelet transfusion–associated risks of IVH and mortality varied according to PC and MPV levels at the time of transfusion.

**Meaning:**

These findings suggest that a lower platelet transfusion threshold is preferred, while the risk of a low PC should not be neglected.

## Teaser

This cohort study evaluates the associations of platelet transfusion, platelet count, and mean platelet volume with intraventricular hemorrhage and in-hospital mortality among preterm infants in China.

## Introduction

Thrombocytopenia, defined as a platelet count (PC) less than 150 × 10^3^/μL, affects 1% to 5% of neonates and occurs in 20% to 50% of neonates in neonatal intensive care units (NICUs) (to convert PC to ×10^9^/L, multiply by 1).^1,2,3,4^ Major bleeding occurs in approximately 5% to 15% of neonates with severe thrombocytopenia in NICUs, and the most important and devastating bleeding event is intraventricular hemorrhage (IVH).^5^

Platelet transfusions are commonly administered to correct severe neonatal thrombocytopenia.^4,5^ In the US, 23% of preterm infants with thrombocytopenia received platelet transfusion.^3^ However, whether these transfusions are beneficial or detrimental is still controversial. Evidence suggests that platelet transfusion is associated with increased mortality and comorbidities, including IVH, among preterm infants.^3,4^ The results of the PlaNet-2 trial^6^ indicated that a more conservative platelet transfusion threshold of 25 × 10^3^/μL was associated with decreased risks of major bleeding and mortality in neonates compared with a threshold of 50 × 10^3^/μL. This finding has undoubtedly informed neonatal platelet transfusion guidelines in many countries, including China.^7,8^ Nevertheless, the results from a retrospective cohort study^9^ indicated that platelet transfusion is not associated with IVH in very-low-birth-weight infants. Variation in the hemostatic system between different ethnic groups was found, but the practice of platelet transfusion in China has been scarcely reported.^10,11^

A certain PC level is commonly used as a criterion for platelet transfusion. Severe thrombocytopenia is considered a potential risk factor for bleeding, although their association has never been convincingly confirmed.^2,5,12^ Except in the context of PC, limited research has investigated whether platelet indices, such as the mean platelet volume (MPV), platelet distribution width (PDW), or platelet–large cell ratio (P-LCR), are associated with IVH and mortality in preterm infants.^2^ Additionally, most current research has focused on the main association of PC or transfusion with IVH or mortality, and whether this platelet transfusion–associated risk varies with PC or other platelet index levels at the time of transfusion has rarely been investigated. This study has 3 objectives: (1) to characterize platelet transfusion practices in preterm infants at one center in China, (2) to evaluate the associations of platelet transfusion, PC, and MPV with IVH and in-hospital mortality among preterm infants, and (3) to explore whether the platelet transfusion–associated risks of IVH and mortality vary based on the PC or MPV level at the time of transfusion.

## Methods

### Study Design and Data Source

This retrospective cohort study was conducted among infants admitted between May 2016 and October 2017 who received any period of mechanical ventilation support during their hospital stay in the Department of Neonatology, Faculty of Pediatrics of the Seventh Medical Center of PLA General Hospital, which serves as a NICU referral center in Beijing, China. All infants were transferred to our center on or after the day of birth. Because their medical records before transfer could be incomplete, infants transferred later than the day after birth were excluded. Term infants with a gestational age (GA) greater than or equal to 37 weeks were also excluded. Eligible infant demographic characteristics (including maternal information), admission records, perinatal records, laboratory test results, diagnostic records (including imaging results), and prescription records were retrieved from their electronic medical records.

This research was approved by the ethics committee of the Seventh Medical Center of PLA General Hospital. Informed consent was waived by the ethics committee because all personal information were deidentified in our retrieved data. This study followed the Strengthening the Reporting of Observational Studies in Epidemiology (STROBE) reporting guidelines.

### Exposures

Platelet transfusion records were retrieved from prescription records. Apheresis platelets in plasma were used. In our center, a blood test is performed daily for preterm infants receiving ventilation in their first 3 days after birth and then twice a week as routine checks. If a decreasing PC trend is observed, subsequent tests will be performed in 8- to 12-hour increments until an increasing PC trend is observed. The infants’ PC results during their hospital stay, along with other platelet index results, including MPV, PDW, and P-LCR, were retrieved from laboratory test records. Methods for measuring PC and MPV were presented in the eMethods in the Supplement.

### Outcomes

In our center, cranial ultrasonography examination is routinely performed in infants as early as possible within 72 hours after admission. In this study, if any sign of IVH was detected, cranial ultrasonography was reperformed at a frequency of at least once a week. An IVH event was defined and graded according to Papile et al.^13^ Severe IVH (grade 3 or 4) was defined as IVH with ventricular dilatation or parenchymal hemorrhage. The grade of IVH events was determined independently and agreed on by 2 neonatologists (C.C. and J.C.) by reviewing all study infants’ cranial ultrasonography or computed tomography records during their hospital stay. An experienced imaging physician was invited to make a final adjudication when discrepant gradings occurred. Time to any grade of IVH was defined as the time from birth to the first occurrence of any grade of IVH, and time to severe IVH was defined as the time from birth to the first occurrence of grade 3 or 4 IVH.

A large proportion of preterm infants in China are discharged against medical advice.^14^ In our study, the end of follow-up was defined as 72 hours after discharge. Time to in-hospital mortality was defined as the time from birth to death from any cause to 72 hours after discharge. Infants confirmed alive at the end of follow-up were censored.

### Covariates

Infant baseline demographic characteristics, perinatal information, and maternal information were collected. Congenital disorders were defined as those that might cause an increased risk of bleeding or death. Sepsis was defined as a positive blood culture. Necrotizing enterocolitis (NEC) was defined as stage 2A or higher, according to the modified Bell staging criteria.^15^

### Statistical Analysis

For any grade IVH or severe IVH, the Fine-Gray subdistribution hazard model was used to account for the competing risk of death.^16^ For in-hospital mortality, the Cox proportional hazards model with the Efron method of handling ties was applied. Platelet transfusion times, PC, and MPV were included in the aforementioned models as time-varying covariates. Sepsis and NEC were combined into one variable (sepsis/NEC). Sepsis/NEC and ventilation were treated as time-varying covariates. PC and MPV were defined as the most recent test results at least 1 day prior to the outcome event to best avoid temporal ambiguity of test result and outcome event. The platelet transfusion number was defined as the cumulative number of platelet transfusions received by an infant 1 day prior to the outcome event to avoid temporal ambiguity as much as possible. Considering that multiple blood test results were available within 24 hours prior to the outcome event and there was a high variance in these results over time, the nadir PC result within 24 hours before the outcome event was selected. For MPV, the highest value was selected. Only linear terms of platelet transfusion number, PC, and MPV were considered when studying their main associations with IVH and mortality. When PC was included as a continuous covariate in the model, its hazard ratio (HR) was minuscule at every 1 × 10^3^/μL decrement, so this HR was converted to a 50 × 10^3^/μL decrement for better explanation. To explore whether the platelet transfusion–associated risks of IVH and mortality depend on the PC or MPV level, interaction terms between platelet transfusion number, PC, and MPV were added. Quadratic and cubic terms of PC were added whenever they were statistically significant. Other covariates were adjusted in the models if they were considered potential confounders according to current evidence.

For secondary analysis, continuous PC was replaced with binary thrombocytopenia, and MPV was also replaced with PDW or P-LCR. The proportion of missing values was small, so complete-case analyses were performed. The significance level was set at *P* < .05, and all tests were 2-sided. All analyses were conducted using SAS version 9.4 (SAS Institute).

## Results

### Patient Characteristics

The study population included 1221 ventilated preterm neonates; 731 (59.9%) were male (Table 1 and eFigure 6 in the Supplement). The median (IQR) birth weight was 1575 (1250-1940) g, and the median (IQR) gestational age was 31 (29-33) weeks. A total of 452 neonates (37.0%) developed thrombocytopenia, and 94 (7.7%) received at least 1 platelet transfusion during their hospital stay. Overall, 263 neonates (21.5%) and 81 neonates (6.6%) developed any grade of IVH and severe IVH, respectively, and 80 neonates (6.6%) died.

**Table 1.** Characteristics of the Study Population by Thrombocytopenia and Reception of Platelet Transfusion

Characteristic	Neonates, No./total No. (%)					
	Overall (N = 1221)	By thrombocytopenia^a^			By platelet transfusion	
		With (n = 452)	Without (n = 767)		Received ≥1 (n = 94)	Received 0 (n = 1127)
Sex						
Male	731/1221 (59.9)	286/452 (63.3)		445/767 (58.0)	56/94 (59.6)	675/1127 (59.9)
Female	490/1221 (40.1)	166/452 (36.7)		322/767 (42.0)	38/94 (40.4)	452/1127 (40.1)
Weight at birth, g						
Median (IQR)	1575.0 (1250.0-1940.0)	1300.0 (1060.0-1565.0)		1730.0 (1430.0-2112.0)	1060.0 (930.0-1290.0)	1600.0 (1305.0-1980.0)
Strata						
<1000	95/1221 (7.8)	73/452 (16.2)		22/767 (2.9)	32/94 (34.0)	63/1127 (5.6)
1000-1499	442/1221 (36.2)	242/452 (53.5)		200/767 (26.1)	54/94 (57.4)	388/1127 (34.4)
1500-2499	610/1221 (50.0)	128/452 (28.3)		481/767 (62.71)	6/94 (6.4)	604/1127 (53.6)
2500-3999	74/1221 (6.1)	9/452 (2.0)		64/767 (8.3)	2/94 (2.1)	72/1127 (6.4)
Gestational age						
Median (IQR), wk	31.0 (29.0-33.0)	30.0 (28.0-31.0)		31.0 (30.0-33.0)	28.0 (27.0-30.0)	31.0 (29.0-33.0)
Strata						
<28 wk 0 d	103/1221 (8.4)	73/452 (16.2)		30/767 (3.9)	36/94 (38.3)	67/1127 (5.9)
28 wk 0 d to 31 wk 6 d	663/1221 (54.3)	282/452 (62.4)		381/767 (49.7)	52/94 (55.3)	611/1127 (54.2)
32 wk 0 d to 33 wk 6 d	283/1221 (23.2)	60/452 (13.3)		222/767 (28.9)	0/94 (0)	283/1127 (25.1)
34 wk 0 d to 36 wk 6 d	172/1221 (14.1)	37/452 (8.2)		134/767 (17.5)	6/94 (6.4)	166/1127 (14.7)
Congenital disorder	147/1221 (12.1)	62 (13.7)		85/767 (11.1)	20/94 (21.3)	127/1127 (11.3)
Singleton	990/1221 (81.1)	353 (78.1)		635/767 (82.8)	72/94 (76.6)	918/1127 (81.5)
Apgar score ≤7						
1-min	316/1215 (26.0)	172/450 (38.2)		142/763 (18.6)	53/94 (56.4)	263/1121 (23.5)
5-min	123/1214 (10.1)	68/450 (15.1)		54/762 (7.1)	22/94 (23.4)	101/1120 (9.0)
10-min	69/1202 (5.7)	44/448 (9.8)		24/752 (3.2)	12/93 (12.9)	57/1109 (5.1)
Cesarean delivery	799/1221 (65.4)	297/452 (65.7)		500/767 (65.2)	52/94 (55.3)	747/1127 (66.3)
Maternal preeclampsia	183/1221 (15.0)	109/452 (24.1)		74/767 (9.6)	28/94 (29.8)	155/1127 (13.8)
Pregnancy-induced hypertension	50/1221 (4.1)	27/452 (6.0)		23/767 (3.0)	6/94 (6.4)	44/1127 (3.9)
Antenatal steroids given	745/1221 (61.6)	261/452 (58.1)		484/767 (63.7)	57/94 (62.0)	688/1127 (61.5)
Intrauterine distress	55/1221 (4.5)	32/452 (7.1)		23/767 (3.0)	5/94 (5.3)	50/1127 (4.4)
Maternal age, median (IQR), y	31.0 (28.0-35.0)	32.0 (28.0-35.0)		31.0 (28.0-35.0)	33.0 (29.0-36.0)	31.0 (28.0-35.0)
High-risk pregnancy^b^	290/1221 (23.8)	124/452 (27.4)		166/767 (21.6)	33/94 (35.1)	257/1127 (22.8)
Premature rupture of membrane	224/1221 (18.4)	49/452 (10.8)		175/767 (22.8)	3/94 (3.2)	221/1127 (19.6)
Ventilated later than birthday	70/1221 (5.7)	24/452 (5.3)		46/767 (6.0)	7/94 (7.4)	63/1127 (5.6)
Sepsis^c^	51/1221 (4.2)	38/452 (8.4)		13/767 (1.7)	16/94 (17.0)	35/1127 (3.1)
NEC^d^	6/1221 (0.5)	2/452 (0.4)		4/767 (0.5)	1/94 (1.1)	5/1127 (0.4)
Length of hospital stay, median (IQR), d	35.0 (19.0-52.0)	48.0 (27.5-70.0)		28.0 (16.0-44.0)	66.5 (31.0-93.0)	33.0 (18.0-50.0)
Any grade of IVH	263/1221 (21.5)	156/452 (34.5)		107/767 (14.0)	50/94 (53.2)	213/1127 (18.9)
Grade 3 or 4 IVH	81/1221 (6.6)	61/452 (13.5)		20/767 (2.6)	26/94 (27.7)	55/1127 (4.9)
In-hospital mortality	80/1221 (6.6)	55/452 (12.2)		23/767 (3.0)	18/94 (19.2)	62/1127 (5.5)

Abbreviations: IVH, intraventricular hemorrhage; NEC, necrotizing enterocolitis.

^a^ Thrombocytopenia was defined as any platelet count less than 150 × 10^3^/μL during hospital stay. Results for 2 infants were missing because they did not receive any blood test during hospital stay. Percentages may not add to 100% due to rounding.

^b^ High-risk pregnancy was defined as maternal age less than 17 or greater than 35 years old.

^c^ Sepsis was defined by a positive blood culture.

^d^ NEC was defined as stage 2A or higher, according to modified Bell staging criteria.

### Platelet Transfusion Practice

A total of 166 platelet transfusions were performed in 94 preterm neonates (7.7%). Among them, 63 (67.0%) received transfusions only once. The detailed distribution of transfusion number is presented in eTable 1 in the Supplement.

**Figure 1.**
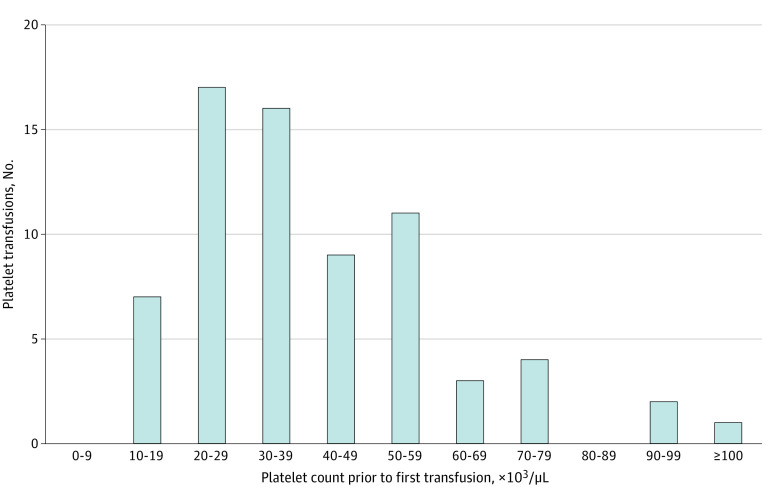
Distribution of Platelet Counts Prior to First Platelet Transfusion in Preterm Infants Infants who received prophylactic transfusions (a transfusion that occurred before any intraventricular hemorrhage) and with platelet count results within 24 hours prior to the transfusion were selected. If multiple platelet count records existed within 24 hours prior to transfusion, the lowest value was selected. To convert platelet count to ×10^9^ per liter, multiply by 1.

In Figure 1, the distribution of PC levels prior to the first platelet transfusion is presented, where nadir PC within 24 hours before the first platelet transfusion was selected, if available. A total of 70 eligible records were finally selected with a median (IQR) PC of 36 × 10^3^/μL (26 × 10^3^/μL to 51 × 10^3^/μL).

### Association of Platelet Transfusion, PC, and MPV With IVH and Mortality

#### Platelet Transfusion

In model 1 (Table 2), platelet transfusion was significantly associated with both IVH outcomes and in-hospital mortality before adjustment for covariates. After adjustment for covariates, PC, and MPV in models 2 and 3, every additional platelet transfusion event was significantly associated with in-hospital mortality. In model 3, every additional platelet transfusion event was associated with an increased the risk of death by nearly 50% (HR, 1.48; 95% CI, 1.13-1.93; *P* = .004). The full results of models 2 and 3 appear in eTable 2 in the Supplement. In the secondary analyses where PC was replaced with thrombocytopenia or MPV was replaced with PDW or P-LCR, this result remained unchanged (eTables 3 and 4 in the Supplement).

**Table 2.** Association of Platelet Transfusion, Platelet Count, and Mean Platelet Volume, With the Risk of IVH and In-Hospital Mortality

Outcome and exposure^c^	Unadjusted model^a^		Adjusted models^b^			
	Model 1		Model 2		Model 3	
	HR (95% CI)	*P* value	HR (95% CI)	*P* value	HR (95% CI)	*P* value
Any grade of IVH						
Platelet transfusion	1.26 (1.04-1.54)	.02	1.11 (0.92-1.35)	.27	1.11 (0.92-1.34)	.28
Platelet count, per 50 × 10^3^/μL decrease	1.18 (1.10-1.26)	<.001	1.12 (1.04-1.21)	.002	1.13 (1.05-1.22)	.001
Mean platelet volume, per 1 μm^3^ increase	1.15 (1.00-1.33)	.05	NA	NA	0.94 (0.82-1.09)	.41
Grade 3 or 4 IVH						
Platelet transfusion	1.75 (1.28-2.41)	<.001	1.28 (0.89-1.84)	.18	1.30 (0.90-1.88)	.16
Platelet count, per 50 × 10^3^/μL decrease	1.36 (1.20-1.54)	<.001	1.17 (1.03-1.33)	.02	1.16 (1.02-1.32)	.02
Mean platelet volume, per 1 μm^3^ increase	1.34 (1.04-1.73)	.02	NA	NA	0.94 (0.73-1.22)	.66
In-hospital mortality						
Platelet transfusion	2.35 (1.88-2.92)	<.001	1.47 (1.13-1.91)	.004	1.48 (1.13-1.93)	.004
Platelet count, per 50 × 10^3^/μL decrease	1.92 (1.67-2.20)	<.001	1.70 (1.45-1.99)	<.001	1.74 (1.48-2.03)	<.001
Mean platelet volume, per 1 μm^3^ increase	1.20 (0.93-1.55)	.17	NA	NA	0.83 (0.69-0.98)	.03

Abbreviations: IVH, intraventricular hemorrhage; HR, hazard ratio; NA, not applicable.

SI conversion factor: To convert platelet count to ×10^9^ per liter, multiply by 1.

^a^ Platelet transfusion, platelet count, and mean platelet volume were separately included in model 1 as the only time-varying variable.

^b^ In model 2, platelet transfusion and platelet count were simultaneously included as time-varying variables. Additionally, infant sex, birth weight strata, gestational age strata, congenital disorder, singleton, any Apgar score of 7 or less (1-minute, 5-minute, or 10-minute Apgar), cesarean delivery, maternal preeclampsia, pregnancy-induced hypertension, antenatal steroids given, intrauterine distress, maternal high-risk pregnancy, and premature rupture of membrane were adjusted as time-constant covariates. First ventilation time after birth and the sepsis/necrotizing enterocolitis occurrence time were adjusted as time-varying covariates. In model 3, mean platelet volume was added to model 2 as a time-varying covariate.

^c^ For any grade of IVH and grade 3 or 4 IVH outcomes, the Fine-Gray subdistribution hazard model was used to account for the competing risk of death. For in-hospital mortality outcome, the Cox proportional hazards model was used. *P* values were calculated using corresponding models. The platelet transfusion number was defined as the cumulative number of platelet transfusions received by an infant 1 day prior to the outcome event. The platelet count and mean platelet volume were defined as the most recent results at least 1 day prior to the outcome event.

#### PC

The median (IQR) time from the most recent PC result to the occurrence of both IVH outcomes and mortality was 1 (1-2) day. PC was associated with both IVH outcomes or in-hospital mortality before and after adjustment for covariates. In model 3, every 50 × 10^3^/μL decrement of PC was significantly associated with an increased risk of any grade of IVH (HR, 1.13; 95% CI, 1.05-1.22; *P* = .001), severe IVH (HR, 1.16; 95% CI, 1.02-1.32; *P* = .02), and mortality (HR, 1.74; 95% CI, 1.48-2.03; *P* < .001). These results remained similar after MPV was replaced with PDW or P-LCR in model 3. When PC was replaced with thrombocytopenia as a binary variable in model 3 (eTable 3 in the Supplement), thrombocytopenia was significantly associated with any grade IVH (HR, 1.69; 95% CI, 1.24-2.31; *P* < .001), severe IVH (hazard ratio, 2.02; 95% CI, 1.18-3.46; *P* = .02), and mortality (hazard ratio, 6.45; 95% CI, 3.79-10.99; *P* < .001).

#### MPV

The median (IQR) times from the most recent MPV result to the occurrence of any grade IVH, severe IVH, and mortality were 1 (1-3) days, 2 (1-3) days, and 2 (1-4) days, respectively. In model 3, every percentage increase in MPV was significantly associated with a 17% decreased risk of mortality (hazard ratio, 0.83; 95% CI, 0.69-0.98; *P* = .03).

### Association Between PC, MPV, and Platelet Transfusion–Associated Risk of IVH and Mortality

#### PC

**Figure 2.**
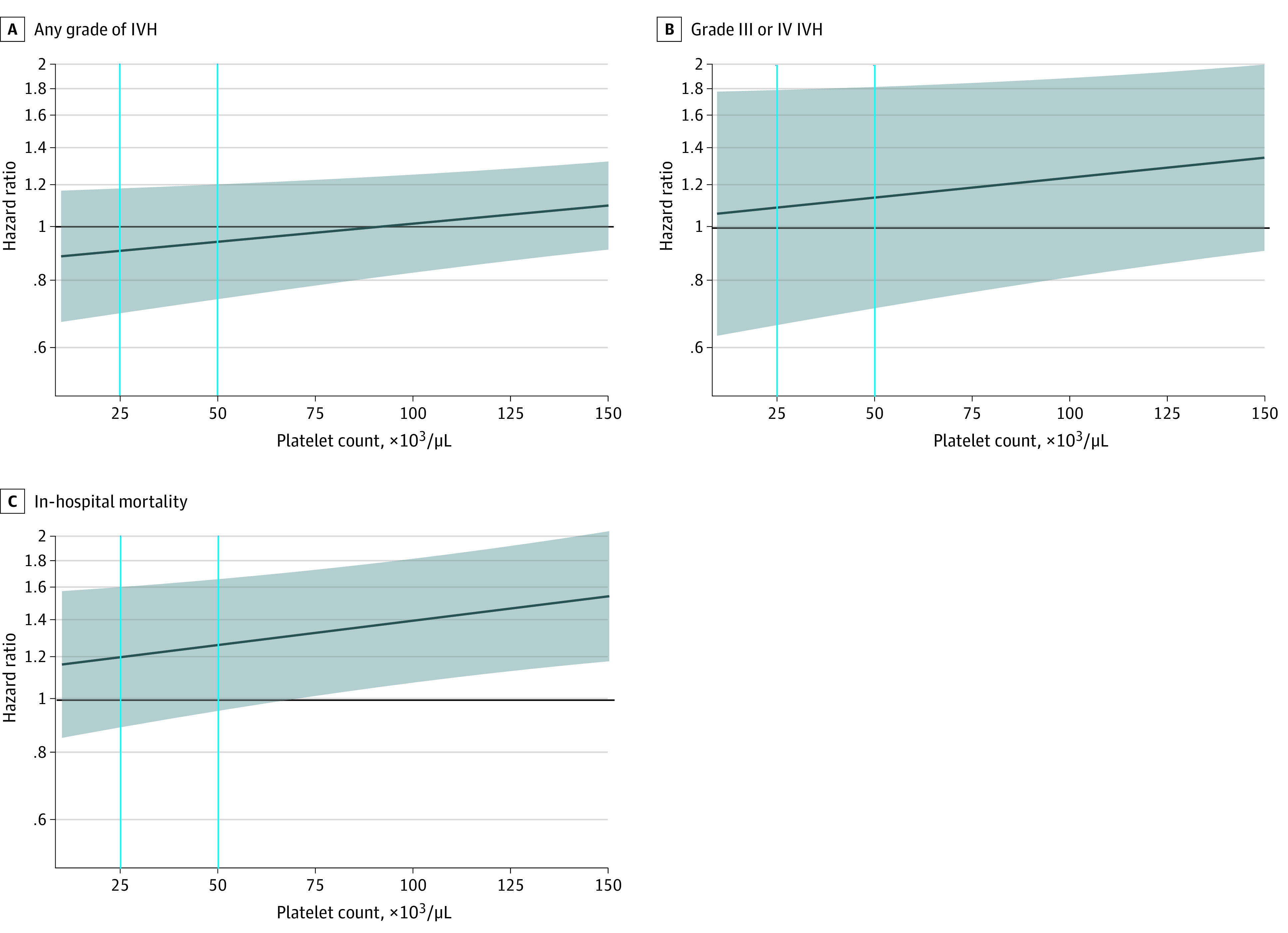
Association Between Platelet Transfusion–Associated Outcomes and Platelet Count at the Time of Platelet Transfusion The dark blue line represents the hazard ratio, and the shaded area represents the 95% CI. The horizontal black line indicates a hazard ratio of 1.00. Vertical lines indicate platelet count values of 25 × 10^3^ /μL and 50 × 10^3^/μL (to convert to ×10^9^ per liter, multiply by 1). A and B, Hazard ratios were estimated by the Fine-Gray subdistribution hazard model to account for the competing risk of death. C, The Cox proportional hazards model was used to estimate the hazard ratio. In all models, platelet count was included as a time-varying variable, which was defined as the most recent result at least 1 day prior to the outcome event. The interaction term between platelet count and platelet transfusion was included in all models, so the hazard ratio represents the risk change for every additional platelet transfusion at a certain platelet count level. The quadratic term of platelet count was added to the model of any grade IVH, and quadratic and cubic terms were added to the model of in-hospital mortality because they were statistically significant. Infant sex, birth weight strata, gestational age strata, congenital disorder, singleton, any Apgar score of 7 or lower (ie, 1-minute, 5-minute, or 10-minute Apgar), cesarean delivery, maternal preeclampsia, pregnancy-induced hypertension, antenatal steroids given, intrauterine distress, maternal high-risk pregnancy, and premature rupture of membrane were adjusted as time-constant covariates; first ventilation time after birth and the sepsis/necrotizing enterocolitis occurrence time after birth were adjusted as time-varying covariates.

In Figure 2, the platelet transfusion–associated hazard ratio of each outcome was plotted against the PC level at the time of transfusion. The platelet transfusion–associated risk of either IVH outcome or mortality varied with PC level. For example, the platelet transfusion–associated HR for mortality was 1.20 (95% CI, 0.89-1.62) when the PC level was 25 × 10^3^/μL at the time of transfusion, but this became 1.26 (95% CI, 0.96-1.67) at a PC level of 50 × 10^3^/μL and 1.40 (95% CI, 1.08-1.82) at a PC level of 100 × 10^3^/μL. Extension to higher PC levels and numerical estimates of HRs are presented in eTable 5 and eFigure 1 in the Supplement. Survival plots grouped by pretransfusion platelet count at a cutoff point of 25 × 10^3^/μL or 50 × 10^3^/μL are presented in eFigures 4 and 5 in the Supplement.

#### MPV

**Figure 3.**
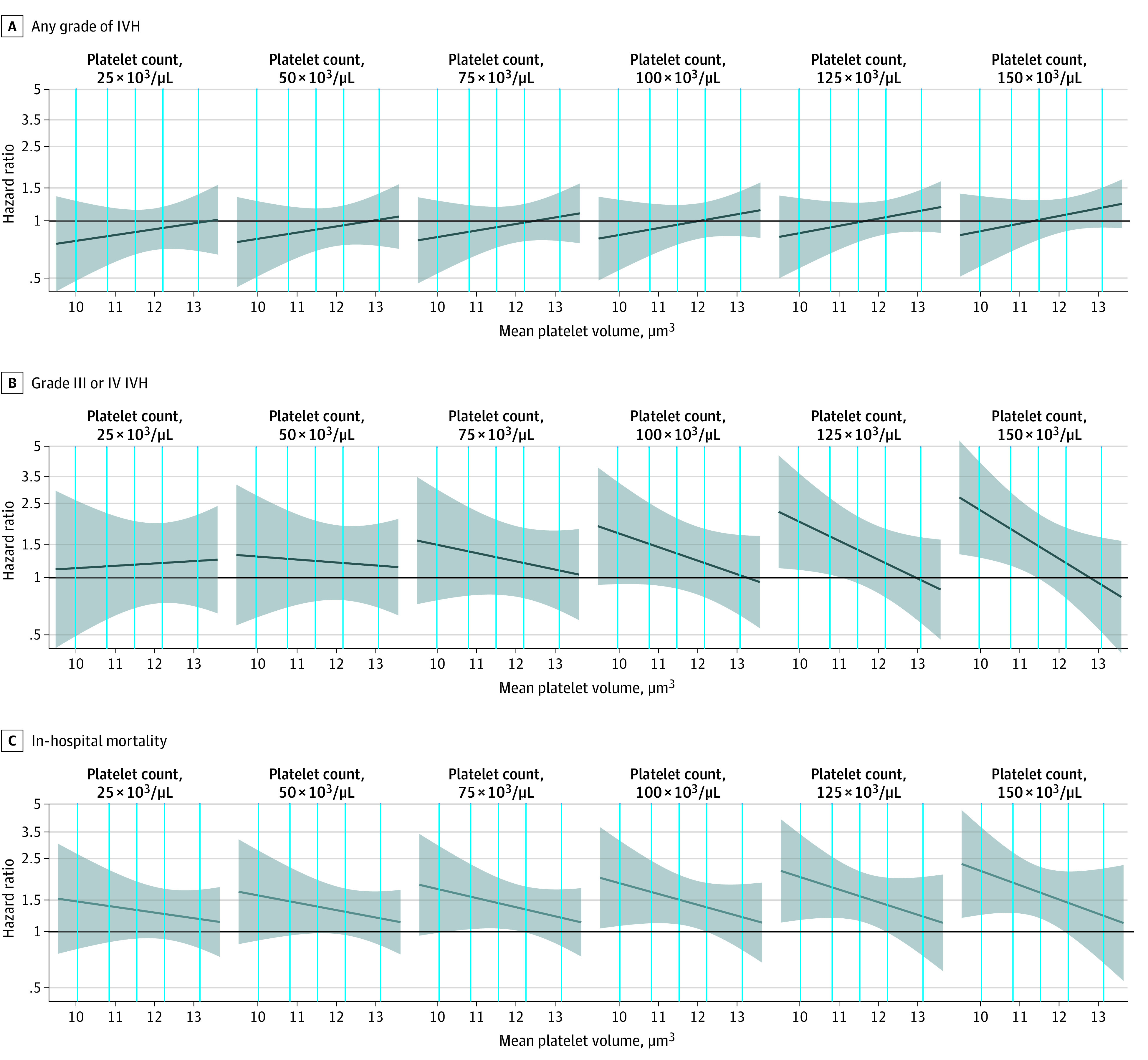
Association Between Platelet Transfusion–Associated Outcomes and Mean Platelet Volume at the Time of Platelet Transfusion The dark blue line represents the hazard ratio, and the shaded area represents the 95% CI. The black horizontal line indicates a hazard ratio of 1.00. Vertical lines indicate the 5th, 25th, 50th, 75th, and 95th percentiles of mean platelet volume records in the whole sample. A and B, Hazard ratios were estimated by the Fine-Gray subdistribution hazard model to account for the competing risk of death. C, The Cox proportional hazards model was used to estimate the hazard ratio. In all models, platelet count and mean platelet volume were included as time-varying variables, which were defined as the most recent test result at least 1 day prior to the outcome event. Additionally, infant sex, birth weight strata, gestational age strata, congenital disorder, singleton birth, any Apgar score of 7 or lower (ie, 1-minute, 5-minute, or 10-minute Apgar), cesarean delivery, maternal preeclampsia, pregnancy-induced hypertension, antenatal steroid use, intrauterine distress, maternal high-risk pregnancy, and premature rupture of membrane were adjusted as time-constant covariates. First ventilation time after birth and the sepsis/necrotizing enterocolitis occurrence time after birth were adjusted as time-varying covariates. In addition, 2-way interaction terms between platelet count and platelet transfusion and between mean platelet volume and platelet transfusion as well as 3-way interaction terms between platelet count, mean platelet volume, and platelet transfusion were included in all models. Despite the linear term, the quadratic term of platelet count was added to the model of any grade intraventricular hemorrhage, and quadratic and cubic terms were added to the model of in-hospital mortality.

In Figure 3, the platelet transfusion–associated HR of each outcome was plotted against MPV levels at transfusion at 6 PC levels, ranging from 25 × 10^3^/μL to 150 × 10^3^/μL; for detailed numeric results, see eTable 6 in the Supplement. The association between the platelet transfusion–associated HR of either severe IVH or mortality and the MPV level varied with the level of PC at the time of platelet transfusion. The platelet transfusion–associated HR for either severe IVH or mortality with the MPV level seemed to become more severe as the PC level increased. For example, supposing the transfusion was performed at a PC level of 50 × 10^3^/μL, the platelet transfusion–associated risk would range from 1.31 (95% CI, 0.62-2.77) for an MPV of 10.0 μm^3^ to 1.17 (95% CI, 0.70-1.95) for an MPV of 13.1 μm^3^. However, this range became 2.01 (95% CI, 1.11-3.64) to 0.98 (95% CI, 0.58-1.65) at a PC level of 125 × 10^3^/μL. These trends were similar for PDW and P-LCR (eFigures 2 and 3 in the Supplement).

## Discussion

In our study, 7.7% of preterm infants receiving ventilation also received platelet transfusion, which was close to 9.4%, the proportion of infants receiving 1 or more platelet transfusion in US NICUs.^17^ Half of those who received a platelet transfusion first received platelet transfusion at a PC of less than 36 × 10^3^/μL, which was between 27 × 10^3^/μL, as reported in the United Kingdom, and approximately 50 × 10^3^/μL, as reported in the United States.^9^

There is no lack of research investigating the associations of platelet transfusion, PC, and bleeding with mortality among neonates.^2^ However, few studies have examined these factors simultaneously. In our study, platelet transfusion, PC, and MPV were evaluated simultaneously as time-varying covariates, while other potential confounding factors were adjusted.

In our study, platelet transfusion was significantly associated with mortality, and every additional transfusion was associated with a 0.5-fold increase in mortality risk among preterm infants. Excessive platelet transfusion has long been considered a risk factor for death among neonates.^4,17,18^ In a cross-sectional study conducted in premature infants in the United States, platelet transfusion was associated with not only increased mortality but also comorbidities, including IVH.^3^

However, in our study, an association between platelet transfusion and IVH was not found. In another retrospective cohort study,^9^ the association between platelet transfusion and severe IVH disappeared after adjustment for the pretransfusion nadir PC level in very low-birth-weight infants.^9^

Although platelet transfusion is the hallmark of treatment for severe neonatal thrombocytopenia, it has long been disputed whether PC is associated with bleeding or death among preterm infants.^5^ In a previous study,^19^ an association between nadir PC and major bleeding was not observed in a cohort of neonates with severe thrombocytopenia. In another retrospective cohort study,^9^ thrombocytopenia was observed to be a risk factor for IVH, but the severity of thrombocytopenia was not. In our study, however, a lower PC or thrombocytopenia was significantly associated with increased risks of IVH and mortality. The PC can decrease quickly without being detected, and IVH might induce no symptoms as well.^2^ Thus, the nadir PC prior to IVH does not necessarily reflect neonates’ actual PC status before IVH, and the temporal sequence of PC time and IVH time is therefore difficult to determine. IVH is a multifactorial process, and a variety of confounders need to be adjusted for.^5^ In our study, these difficulties were considered by treating PC as a time-varying dependent variable in the multivariable survival analysis model, and PC results at least 1 day prior to the diagnostic day of IVH were selected. In another study in which PC was treated as a time-varying covariate,^20^ it was a significantly associated with major bleeding in preterm neonates with thrombocytopenia.^20^

The MPV reflects platelet size. An increased MPV is suggestive of increasing platelet production and activation. Larger platelets also contain more granules and prothrombotic materials.^21^ Whether the initial MPV value can be prognostic of mortality in critically ill adult patients remains unclear.^21^ The role of MPV in this process is even more puzzling in neonates. It has been reported that high MPV in the first hours of life may be associated with IVH in extremely preterm infants.^22^ In our study, a higher MPV was found to be independently associated with reduced risk of mortality after adjusting for PC. As neonatal hemostasis is strikingly different from adult hemostasis, further research is warranted.^5^

In the PlaNeT-2 study,^6^ the high-threshold group (50 × 10^3^/μL) had a higher rate of mortality and major bleeding than the low-threshold group (25 × 10^3^/μL). However, it is difficult to conclude whether this result was caused by the different PC levels at the time of transfusion or the different proportions of infants receiving transfusion between the groups (90% in the high-threshold group vs 53% in the low-threshold group received transfusion). In our study, the platelet transfusion–associated risk of both IVH and mortality increased when transfusion was performed at a higher PC level. However, the benefit of transfusing platelets at a lower threshold might be offset by an increased risk of bleeding owing to a decreased PC.

Regardless of the PC, the platelet transfusion–associated risks of severe IVH and mortality varied with the MPV level at transfusion. Adult platelets appear to be more hyperreactive than neonatal platelets.^5,23^ The presence of adult platelets might tilt the neonatal primary hemostatic balance toward a prothrombotic state.^24^ A high MPV in infants might attenuate posttransfusion risks because larger platelets are functionally, metabolically, and enzymatically more active than smaller ones; thus, a hemostatic system with more platelets and a larger portion of larger platelets would probably be more compatible with exogenous adult platelets.^5,21^ If this finding holds true, transfusion might be performed in infants more safely at a higher MPV level before the PC decreases to a certain threshold, and potential comorbidities associated with a decreased PC can thus be avoided.

### Limitations

This study has limitations. Only preterm infants receiving ventilation at a single center were studied, and generalization of our findings may be limited. The sample size was small for detecting interaction terms. The data were collected 5 years ago when platelet transfusion guidelines for newborn infants from the United Kingdom were used in our center, and practices may have been updated since then.^25^ Possible reverse causation, ie, that decreased PC was caused by an asymptomatic bleeding, cannot be excluded.

## Conclusions

In this retrospective cohort study in preterm infants receiving ventilation, platelet transfusion was associated with mortality. Decreased PC levels and thrombocytopenia were associated with increased risks of IVH and mortality. Increased MPV levels were associated with a lower risk of mortality. The platelet transfusion–associated risks of IVH and mortality appeared to vary with the PC and MPV levels at the time of transfusion. More research is needed to confirm and extend our findings.

## Notes

Supplement. **eTable 1. **Distribution of Total Platelet Transfusion Number During Hospital Stay **eTable 2. **Association of Platelet Transfusion, Platelet Count, and Mean Platelet Volume With Risk of IVH and In-Hospital Mortality: Full Results **eTable 3. **Association Between Platelet Transfusion, Thrombocytopenia, Mean Platelet Volume, and Risk of IVH and In-Hospital Mortality **eTable 4. **Association of Platelet Transfusion, Platelet Count, Platelet Distribution Width, or Platelet–Large Cell Ratio With Risk of IVH and In-Hospital Mortality **eTable 5. **Association Between Platelet Transfusion–Associated Outcomes and Platelet Count, With Hazard Ratios at Certain Platelet Count Levels **eTable 6. **Association Between Platelet Transfusion–Associated Outcomes and Mean Platelet Volume, With Hazard Ratios at Certain Combinations of Platelet Count Levels and Mean Platelet Volume Levels **eFigure 1. **Association Between Platelet Transfusion–Associated Outcomes and Platelet Count, Extended **eFigure 2. **Association Between Platelet Transfusion–Associated Outcomes and Platelet Distribution Width **eFigure 3.** Association Between Platelet Transfusion–Associated Outcomes and Platelet–Large Cell Ratio **eFigure 4. **Survival Plots Grouped by Pretransfusion Platelet Count at a Cutoff of 25 × 10^3^/μL **eFigure 5.** Survival Plots Grouped by Pretransfusion Platelet Count at a Cutoff of 50 × 10^3^/μL **eFigure 6.** Flowchart of Selection of the Study Population **eMethods.** Methods for Measuring Platelet Count and Mean Platelet Volumes and Reference Ranges
