# Supplementary material for: Evaluation of the Association of Platelet Count, Mean Platelet Volume, and Platelet Transfusion With Intraventricular Hemorrhage and Death Among Preterm Infants
Source: JAMA Netw Open. 2022 Oct 19;5(10):e2237588. doi: 10.1001/jamanetworkopen.2022.37588 (PMC9582899; doi:10.1001/jamanetworkopen.2022.37588)
Supplement: Supplement. — eTable 1. Distribution of Total Platelet Transfusion Number During Hospital Stay eTable 2. Association of Platelet Transfusion, Platelet Count, and Mean Platelet Volume With Risk of IVH and In-Hospital Mortality: Full Results eTable 3. Association Between Platelet Transfusion, Thrombocytopenia, Mean Platelet Volume, and Risk of IVH and In-Hospital Mortality eTable 4. Association of Platelet Transfusion, Platelet Count, Platelet Distribution Width, or Platelet–Large Cell Ratio With Risk of IVH and In-Hospital Mortality eTable 5. Association Between Platelet Transfusion–Associated Outcomes and Platelet Count, With Hazard Ratios at Certain Platelet Count Levels eTable 6. Association Between Platelet Transfusion–Associated Outcomes and Mean Platelet Volume, With Hazard Ratios at Certain Combinations of Platelet Count Levels and Mean Platelet Volume Levels eFigure 1. Association Between Platelet Transfusion–Associated Outcomes and Platelet Count, Extended eFigure 2. Association Between Platelet Transfusion–Associated Outcomes and Platelet Distribution Width eFigure 3. Association Between Platelet Transfusion–Associated Outcomes and Platelet–Large Cell Ratio eFigure 4. Survival Plots Grouped by Pretransfusion Platelet Count at a Cutoff of 25 × 103/μL eFigure 5. Survival Plots Grouped by Pretransfusion Platelet Count at a Cutoff of 50 × 103/μL eFigure 6. Flowchart of Selection of the Study Population eMethods. Methods for Measuring Platelet Count and Mean Platelet Volumes and Reference Ranges [file jamanetwopen-e2237588-s001.pdf]

Supplemental Online Content

Chen C, Wu S, Chen J, et al. Evaluation of the association of platelet count, mean platelet volume, and platelet transfusion with intraventricular hemorrhage and death among preterm infants. *JAMA Netw Open.* 2022;5(10):e2237588. doi:10.1001/jamanetworkopen.2022.37588

eTable 1. Distribution of Total Platelet Transfusion Number During Hospital Stay

eTable 2. Association of Platelet Transfusion, Platelet Count, and Mean Platelet Volume With Risk of IVH and In-Hospital Mortality: Full Results

eTable 3. Association Between Platelet Transfusion, Thrombocytopenia, Mean Platelet Volume, and Risk of IVH and In-Hospital Mortality

eTable 4. Association of Platelet Transfusion, Platelet Count, Platelet Distribution Width, or Platelet–Large Cell Ratio With Risk of IVH and In-Hospital Mortality

eTable 5. Association Between Platelet Transfusion-Associated Outcomes and Platelet Count, With Hazard Ratios at Certain Platelet Count Levels

eTable 6. Association Between Platelet Transfusion–Associated Outcomes and Mean Platelet Volume, With Hazard Ratios at Certain Combinations of Platelet Count Levels and Mean Platelet Volume Levels

eFigure 1. Association Between Platelet Transfusion–Associated Outcomes and Platelet Count, Extended

eFigure 2. Association Between Platelet Transfusion–Associated Outcomes and Platelet Distribution Width

eFigure 3. Association Between Platelet Transfusion-Associated Outcomes and Platelet–Large Cell Ratio

eFigure 4. Survival Plots Grouped by Pretransfusion Platelet Count at a Cutoff of 25 × 10^3/μL

eFigure 5. Survival Plots Grouped by Pretransfusion Platelet Count at a Cutoff of 50 × 10^3/μL

eFigure 6. Flowchart of Selection of the Study Population

eMethods. Methods for Measuring Platelet Count and Mean Platelet Volumes and Reference Ranges

This supplemental material has been provided by the authors to give readers additional information about their work.

© 2022 Chen C et al. *JAMA Network Open.*

eTable 1. Distribution of Total Platelet Transfusion Number During Hospital Stay

**Total Platelet Transfusion number During Hospital Stay**	**No. of Infants (%)**
1	63/94 (67)
2	14/94 (15)
3	7/94 (7)
4	3/94 (3)
5	2/94 (2)
6	4/94 (4)
8	1/94 (1)
**Total**	**94**

© 2022 Chen C et al. *JAMA Network Open.*

eTable 2. Association of Platelet Transfusion, Platelet Count, and Mean Platelet Volume With Risk of IVH and In-Hospital Mortality: Full Results

		Model 2 [c]			Model 3 [c]	
**Outcomes [a]**	**Covariates [b]**	**HR (95% CI)**	***P* value**		**HR (95% CI)**	***P* value**
Any Grade of IVH	Platelet transfusion	1.11 (0.92-1.35)	.27		1.11 (0.92-1.34)	.28
	Platelet count, per 50 000/µL decrease	1.12 (1.04-1.21)	.002		1.13 (1.05-1.22)	.001
	Mean platelet volume, per 1 μm^3 increase	NA	NA		0.94 (0.82-1.09)	.41
	Sex (female vs. male)	1.05 (0.81-1.35)	.71		1.03 (0.80-1.34)	.81
	Gestational age, wks. (28-31 vs. < 28)	0.63 (0.42-0.94)	.02		0.63 (0.42-0.95)	.03
	Gestational age, wks. (32-33 vs. < 28)	0.29 (0.15-0.54)	<.001		0.29 (0.15-0.54)	<.001
	Gestational age, wks. (34-36 vs. <28)	0.43 (0.20-0.92)	.03		0.41 (0.19-0.88)	.02
	Weight at birth, g (1000-1499 vs. < 1000)	0.94 (0.63-1.38)	.74		0.90 (0.61-1.34)	.62
	Weight at birth, g (1500-2499 vs. < 1000)	0.68 (0.42-1.10)	.12		0.65 (0.40-1.06)	.08
	Weight at birth, g (2500-3999 vs. < 1000)	0.47 (0.14-1.57)	.22		0.47 (0.14-1.60)	.23
	Cesarean delivery (no vs. yes)	1.38 (1.02-1.87)	.04		1.36 (1.00-1.85)	.048
	Maternal preeclampsia (no vs. yes)	1.16 (0.77-1.73)	.48		1.19 (0.79-1.80)	.41
	High-risk pregnancy (no vs. yes)	0.81 (0.61-1.08)	.15		0.81 (0.61-1.08)	.14
	Congenital disorder (no vs. yes)	1.54 (1.02-2.32)	.04		1.52 (1.00-2.31)	.049
	Singletons (no vs. yes)	1.49 (1.12-1.97)	.006		1.48 (1.12-1.97)	.006
	Premature rupture of membrane (no vs. yes)	1.39 (0.92-2.11)	.12		1.39 (0.92-2.11)	.12
	Apgar score ≤ 7 at 1, 5, or 10 min (no vs. yes)	0.74 (0.57-0.95)	.02		0.75 (0.57-0.97)	.03
	Pregnancy induced hypertension (no vs. yes)	0.95 (0.50-1.80)	.88		0.95 (0.50-1.79)	.88
	Antenatal glucocorticosteroids given (no vs. yes)	0.93 (0.72-1.2)	.60		0.93 (0.72-1.20)	.58
	Intrauterine growth restriction (no vs. yes)	1.73 (0.80-3.72)	.16		1.64 (0.74-3.62)	.22
	Ventilation (yes vs. no)	0.52 (0.19-1.42)	.20		0.51 (0.19-1.41)	.20
	Sepsis/NEC (yes vs. no)	1.35 (0.75-2.46)	.32		1.39 (0.77-2.53)	.28
Grade III or IV IVH	Platelet transfusion	1.28 (0.89-1.84)	.18		1.30 (0.90-1.88)	.16
	Platelet count, per 50 000/µL decrease	1.17 (1.03-1.33)	.02		1.16 (1.02-1.32)	.02
	Mean platelet volume, per 1 μm^3 increase	NA	NA		0.94 (0.73-1.22)	.66
	Sex (female vs. male)	0.85 (0.53-1.35)	.49		0.82 (0.51-1.32)	.40
	Gestational age, wks. (28-31 vs. < 28)	0.34 (0.16-0.70)	.004		0.33 (0.16-0.72)	.005
	Gestational age, wks. (32-33 vs. < 28)	0.30 (0.09-0.99)	.048		0.30 (0.09-1.04)	.057
	Gestational age, wks. (34-36 vs. <28)	0.90 (0.16-5.04)	.91		0.72 (0.11-4.77)	.73
	Weight at birth, g (1000-1499 vs. < 1000)	0.65 (0.31-1.36)	.25		0.61 (0.28-1.33)	.22
	Weight at birth, g (1500-2499 vs. < 1000)	0.18 (0.05-0.62)	.006		0.17 (0.05-0.60)	.006
	Weight at birth, g (2500-3999 vs. < 1000)	0.08 (0.00-1.44)	.09		0.09 (0.00-1.92)	.12
	Cesarean delivery (no vs. yes)	0.95 (0.53-1.68)	.85		0.92 (0.51-1.64)	.77
	Maternal preeclampsia (no vs. yes)	2.78 (1.21-6.36)	.02		3.24 (1.27-8.26)	.01
	High-risk pregnancy (no vs. yes)	1.08 (0.60-1.98)	.79		1.06 (0.57-1.95)	.86
	Congenital disorder (no vs. yes)	1.41 (0.68-2.94)	.35		1.38 (0.66-2.89)	.40
	Singletons (no vs. yes)	1.14 (0.63-2.09)	.66		1.14 (0.62-2.12)	.67
	Premature rupture of membrane (no vs. yes)	1.17 (0.52-2.63)	.71		1.14 (0.50-2.63)	.75
	Apgar score ≤ 7 at 1, 5, or 10 min (no vs. yes)	0.38 (0.23-0.62)	<.001		0.38 (0.23-0.64)	<.001
	Pregnancy induced hypertension (no vs. yes)	1.3 (0.30-5.65)	.72		1.28 (0.29-5.59)	.74
	Antenatal glucocorticosteroids given (no vs. yes)	0.76 (0.47-1.24)	.27		0.75 (0.45-1.24)	.26
	Intrauterine growth restriction (no vs. yes)	1.24 (0.42-3.70)	.70		1.15 (0.38-3.44)	.81
	Ventilation (yes vs. no)	0.54 (0.07-4.23)	.56		0.52 (0.07-4.13)	.54
	Sepsis/NEC (yes vs. no)	0.71 (0.16-3.12)	.65		0.75 (0.17-3.35)	.71
In-hospital mortality	Platelet transfusion	1.47 (1.13-1.91)	.004		1.48 (1.13-1.93)	.004
	Platelet count, per 50 000/µL decrease	1.70 (1.45-1.99)	<.001		1.74 (1.48-2.03)	<.001
	Mean platelet volume, per 1 μm^3 increase	NA	NA		0.83 (0.69-0.98)	.03
	Sex (female vs. male)	0.98 (0.61-1.57)	.92		1.00 (0.62-1.61)	.99

© 2022 Chen C et al. *JAMA Network Open.*

		Model 2 [c]			Model 3 [c]	
**Outcomes [a]**	**Covariates [b]**	**HR (95% CI)**	***P* value**		**HR (95% CI)**	***P* value**
	Gestational age, wks. (28-31 vs. < 28)	1.20 (0.60-2.41)	.61		1.20 (0.59-2.42)	.62
	Gestational age, wks. (32-33 vs. < 28)	2.20 (0.76-6.40)	.15		2.21 (0.75-6.49)	.15
	Gestational age, wks. (34-36 vs. <28)	2.88 (0.81-10.19)	.10		2.91 (0.82-10.29)	.10
	Weight at birth, g (1000-1499 vs. < 1000)	0.49 (0.26-0.92)	.03		0.49 (0.26-0.94)	.03
	Weight at birth, g (1500-2499 vs. < 1000)	0.18 (0.07-0.46)	<.001		0.17 (0.06-0.45)	<.001
	Weight at birth, g (2500-3999 vs. < 1000)	0.27 (0.06-1.30)	.10		0.26 (0.05-1.25)	.09
	Cesarean delivery (no vs. yes)	0.68 (0.39-1.20)	.18		0.68 (0.38-1.21)	.19
	Maternal preeclampsia (no vs. yes)	2.13 (1.04-4.34)	.04		2.35 (1.13-4.89)	.02
	High-risk pregnancy (no vs. yes)	1.37 (0.78-2.42)	.28		1.37 (0.77-2.42)	.28
	Congenital disorder (no vs. yes)	0.89 (0.49-1.62)	.70		0.86 (0.47-1.57)	.62
	Singletons (no vs. yes)	1.43 (0.84-2.43)	.19		1.45 (0.85-2.48)	.17
	Premature rupture of membrane (no vs. yes)	1.42 (0.62-3.27)	.41		1.39 (0.60-3.23)	.44
	Apgar score ≤ 7 at 1, 5, or 10 min (no vs. yes)	0.71 (0.43-1.15)	.16		0.72 (0.44-1.17)	.19
	Pregnancy induced hypertension (no vs. yes)	8.58 (0.50-146.98)	.14		8.64 (0.50-148.69)	.14
	Antenatal glucocorticosteroids given (no vs. yes)	1.81 (1.14-2.89)	.01		1.83 (1.14-2.93)	.01
	Intrauterine growth restriction (no vs. yes)	2.38 (0.64-8.87)	.20		2.35 (0.63-8.84)	.21
	Ventilation (yes vs. no)	0.16 (0.04-0.66)	.01		0.16 (0.04-0.65)	.01
	Sepsis/NEC (yes vs. no)	0.38 (0.07-1.99)	.25		0.39 (0.07-2.08)	.27

Abbreviations: IVH, intraventricular hemorrhage; HR, hazard ratio; NA, not applicable; NEC, necrotizing enterocolitis.

[a] For any grade of IVH and grade III or IV IVH outcomes, the Fine-Gray sub distribution hazard model was used to account for the competing risk of death. For in-hospital mortality outcome, Cox proportional hazards model was used. *P* values were calculated using corresponding models.

[b] The platelet transfusion was defined as the cumulative number of platelet transfusions received by an infant one day prior to the outcome event. The platelet count and mean platelet volume were defined as the most recent results at least one day prior to the outcome event. To convert mean platelet volume to fL, multiply by 1.

[c] In Model 2, platelet transfusion and platelet count were simultaneously included as time-varying variables. Additionally, infant sex, birthweight strata, gestational age strata, congenital disorder, singleton, any Apgar score ≤ 7 (1-min, 5-min, or 10-min Apgar), cesarean delivery, maternal preeclampsia, pregnancy-induced hypertension, antenatal steroids given, intrauterine distress, maternal High-risk pregnancy, and premature rupture of membrane were adjusted as time-constant covariates. First ventilation time after birth and the sepsis/necrotizing enterocolitis occurrence time were adjusted as time-varying covariates. In Model 3, mean platelet volume was added to Model 2 as a time-varying covariate.

© 2022 Chen C et al. *JAMA Network Open.*

eTable 3. Association Between Platelet Transfusion, Thrombocytopenia, Mean Platelet Volume, and Risk of IVH and In-Hospital Mortality

		Unadjusted Model [c]			Adjusted Models [d]			
		Model 1			Model 2		Model 3	
**Outcomes [a]**	**Exposures [b]**	**HR (95% CI)**	***P* Value**		**HR (95% CI)**	***P* Value**	**HR (95% CI)**	***P* Value**
Any grade of IVH	Platelet transfusion	1.26 (1.04-1.54)	.02		1.14 (0.96-1.37)	.14	1.15 (0.96-1.38)	.14
	Thrombocytopenia (yes vs. no)	2.24 (1.69-2.97)	<.001		1.73 (1.28-2.35)	<.001	1.69 (1.24-2.31)	<.001
	Mean platelet volume, per 1 μm^3 increase	1.15 (1.00-1.33)	.054		NA	NA	0.96 (0.84-1.11)	.62
Grade III or IV IVH	Platelet transfusion	1.75 (1.28-2.41)	<.001		1.33 (0.94-1.89)	.11	1.35 (0.94-1.94)	.10
	Thrombocytopenia (yes vs. no)	3.82 (2.46-5.95)	<.001		2.10 (1.24-3.56)	.006	2.02 (1.18-3.46)	.01
	Mean platelet volume, per 1 μm^3 increase	1.34 (1.04-1.73)	.02		NA	NA	0.94 (0.73-1.23)	.66
In-hospital mortality	Platelet transfusion	2.35 (1.88-2.92)	<.001		1.70 (1.32-2.20)	<.001	1.73 (1.33-2.24)	<.001
	Thrombocytopenia (yes vs. no)	9.43 (5.92-15.15)	<.001		5.81 (3.44-9.80)	<.001	6.45 (3.79-10.99)	<.001
	Mean platelet volume, per 1 μm^3 increase	1.20 (0.93-1.55)	.17		NA	NA	0.83 (0.67-1.01)	.06

Abbreviations: IVH, intraventricular hemorrhage; HR, hazard ratio; NA, not applicable.

[a] For any grade of IVH and grade III or IV IVH outcomes, the Fine-Gray subdistribution hazard model was used to account for the competing risk of death. For in-hospital mortality outcome, Cox proportional hazards model was used. *P* values were calculated using corresponding models.

[b] The platelet transfusion was defined as the cumulative number of platelet transfusions received by an infant one day prior to the outcome event. The thrombocytopenia was defined as whether the most recent platelet count result at least one day prior to the outcome event was less than 150 000/µL. The mean platelet volume was defined as the most recent results at least one day prior to the outcome event. To convert mean platelet volume to fL, multiply by 1.

[c] Platelet transfusion, thrombocytopenia, or mean platelet volume was separately included in Model 1 as the only time-varying variable.

[d] In Model 2, platelet transfusion and thrombocytopenia were simultaneously included as time-varying variables. Additionally, infant sex, birthweight strata, gestational age strata, congenital disorder, singleton, any Apgar score ≤ 7 (1-min, 5-min, or 10-min Apgar), cesarean delivery, maternal preeclampsia, pregnancy-induced hypertension, antenatal steroids given, intrauterine distress, maternal High-risk pregnancy, and premature rupture of membrane were adjusted as time-constant covariates. First ventilation time after birth and the sepsis/necrotizing enterocolitis occurrence time were adjusted as time-varying covariates. In Model 3, mean platelet volume was added to Model 2 as a time-varying covariate.

© 2022 Chen C et al. *JAMA Network Open.*

eTable 4. Association of Platelet Transfusion, Platelet Count, Platelet Distribution Width, or Platelet–Large Cell Ratio With Risk of IVH and In-Hospital Mortality

		Model 1 (Unadjusted) [c]			Model 2 (Adjusted) [d]	
Outcomes [a]	Exposures [b]	HR (95% CI)	*P* Value [c]		HR (95% CI)	*P* Value [e]
**PDW**						
Any grade of IVH	Platelet transfusion	NA	NA		1.11 (0.92-1.34)	.29
	Platelet count, per 50 000/μL decrease	NA	NA		1.13 (1.05-1.22)	.001
	PDW, per % increase	1.06 (1.01-1.11)	.03		0.98 (0.93-1.03)	.42
Grade III or IV IVH	Platelet transfusion	NA	NA		1.31 (0.91-1.88)	.15
	Platelet count, per 50 000/μL decrease	NA	NA		1.15 (1.01-1.31)	.04
	PDW, per % increase	1.14 (1.07-1.22)	<.001		1.02 (0.94-1.10)	.65
In-hospital mortality	Platelet transfusion	NA	NA		1.47 (1.13-1.92)	.004
	Platelet count, per 50 000/μL decrease	NA	NA		1.75 (1.49-2.06)	<.001
	PDW, per % increase	1.12 (1.05-1.20)	.001		0.96 (0.89-1.04)	.33
**P-LCR**						
Any grade of IVH	Platelet transfusion	NA	NA		1.11 (0.92-1.34)	.28
	Platelet count, per 50 000/μL decrease	NA	NA		1.13 (1.05-1.22)	.001
	P-LCR, per % increase	1.02 (1.00-1.04)	.06		0.99 (0.97-1.01)	.35
Grade III or IV IVH	Platelet transfusion	NA	NA		1.31 (0.90-1.89)	.16
	Platelet count, per 50 000/μL decrease	NA	NA		1.17 (1.03-1.33)	.02
	P-LCR, per % increase	1.04 (1.00-1.08)	.03		0.99 (0.95-1.03)	.58
In-hospital mortality	Platelet transfusion	NA	NA		1.49 (1.14-1.94)	.003
	Platelet count, per 50 000/μL decrease	NA	NA		1.75 (1.49-2.05)	<.001
	P-LCR, per % increase	1.03(1.00-1.07)	.07		0.97 (0.93-1.00)	.07

Abbreviations: IVH, intraventricular hemorrhage; HR, hazard ratio; NA, not applicable; PDW, platelet distribution width; P-LCR, platelet-large cell ratio.

[a] For any grade of IVH and grade III or IV IVH outcomes, the Fine-Gray sub distribution hazard model was used to account for the competing risk of death. For in-hospital mortality outcome, Cox proportional hazards model was used. *P* values were calculated using corresponding models.

[b] The platelet transfusion was defined as the cumulative number of platelet transfusions received by an infant one day prior to the outcome event. The platelet count, PDW, or P-LCR were defined as the most recent results at least one day prior to the outcome event. To convert PDW or P-LCR to proportion of 1.0, multiply by 0.01.

[c] PDW or P-LCR was separately included in Model 1 as the only time-varying variable.

[d] In Model 2, in addition to PDW or P-LCR, infant sex, birthweight strata, gestational age strata, congenital disorder, singleton, any Apgar score ≤ 7 (1-min, 5-min, or 10-min Apgar), cesarean delivery, maternal preeclampsia, pregnancy-induced hypertension, antenatal steroids given, intrauterine

© 2022 Chen C et al. *JAMA Network Open.*

distress, maternal High-risk pregnancy, and premature rupture of membrane were adjusted as time-constant covariates. First ventilation time after birth and the sepsis/necrotizing enterocolitis occurrence time were adjusted as time-varying covariates.

© 2022 Chen C et al. *JAMA Network Open.*

eTable 5. Association Between Platelet Transfusion-Associated Outcomes and Platelet Count, With Hazard Ratios at Certain Platelet Count Levels

	**HR (95% CI) [b]**					
**Outcomes [a]**	**PCT=25 000/µL**	**PCT=50 000/µL**	**PCT=75 000/µL**	**PCT=100 000/µL**	**PCT=125 000/µL**	**PCT=150 000/µL**
Any grade of IVH	0.90 (0.69-1.18)	0.94 (0.74-1.20)	0.98 (0.78-1.22)	1.01 (0.82-1.25)	1.05 (0.87-1.28)	1.09 (0.91-1.32)
Grade III or IV IVH	1.09 (0.66-1.80)	1.14 (0.71-1.82)	1.19 (0.76-1.85)	1.24 (0.81-1.89)	1.29 (0.86-1.94)	1.35 (0.91-2.00)
In-hospital mortality	1.20 (0.89-1.62)	1.26 (0.96-1.67)	1.33 (1.02-1.74)	1.40 (1.08-1.82)	1.48 (1.13-1.93)	1.56 (1.18-2.05)

Abbreviations: PCT, platelet count; HR, hazard ratio.

[a] For any grade of IVH and grade III or IV IVH outcomes, the Fine-Gray sub distribution hazard model was used to account for the competing risk of death. For in-hospital mortality outcome, Cox proportional hazards model was used. *P* values were calculated using corresponding models.

[b] In models of all three outcomes, the platelet count was included as a time-varying variable and was defined as the most recent test result at least one day prior to the outcome event. Interaction term between platelet count and platelet transfusion was included in all three models, so the hazard ratio represented risk change for every additional platelet transfusion at certain platelet count levels. Despite of linear term, quadratic term of platelet count was added to the model of any grade of IVH, and quadratic and cubic terms were added to the model of in-hospital mortality since they were statistically significant. In all three models, infant sex, birthweight strata, gestational age strata, congenital disorder, singleton, any Apgar score ≤ 7 (1-min, 5-min, or 10-min Apgar), cesarean delivery, maternal preeclampsia, pregnancy-induced hypertension, antenatal steroids given, intrauterine distress, maternal High-risk pregnancy, and premature rupture of membrane were adjusted as time-constant covariates; first ventilation time after birth and the sepsis/necrotizing enterocolitis occurrence time after birth were adjusted as time-varying covariates.

© 2022 Chen C et al. *JAMA Network Open.*

eTable 6. Association Between Platelet Transfusion–Associated Outcomes and Mean Platelet Volume, With Hazard Ratios at Certain Combinations of Platelet Count Levels and Mean Platelet Volume Levels

		HR (95% CI) [c]					
**Outcomes [a]**	**MPV Level, µm³ [b]**	**PCT=25 000/µL**	**PCT=50 000/µL**	**PCT=75 000/µL**	**PCT=100 000/µL**	**PCT=125 000/µL**	**PCT=150 000/µL**
Any grade of IVH	10.0	0.79 (0.48-1.29)	0.81 (0.51-1.28)	0.83 (0.53-1.29)	0.85 (0.55-1.31)	0.87 (0.56-1.33)	0.89 (0.58-1.37)
	10.3	0.81 (0.52-1.25)	0.83 (0.55-1.25)	0.85 (0.57-1.26)	0.87 (0.59-1.28)	0.89 (0.60-1.31)	0.91 (0.62-1.35)
	10.8	0.84 (0.59-1.19)	0.86 (0.61-1.20)	0.88 (0.64-1.22)	0.91 (0.66-1.25)	0.93 (0.68-1.28)	0.96 (0.69-1.32)
	11.5	0.88 (0.67-1.16)	0.90 (0.70-1.17)	0.93 (0.73-1.20)	0.96 (0.75-1.22)	0.99 (0.78-1.26)	1.02 (0.80-1.30)
	12.2	0.92 (0.71-1.20)	0.96 (0.75-1.22)	0.99 (0.79-1.24)	1.02 (0.82-1.27)	1.05 (0.86-1.30)	1.09 (0.89-1.34)
	12.8	0.96 (0.91-1.31)	1.00 (0.75-1.33)	1.04 (0.80-1.35)	1.07 (0.84-1.37)	1.11 (0.88-1.40)	1.15 (0.93-1.44)
	13.1	0.99 (0.70-1.40)	1.02 (0.74-1.41)	1.06 (0.79-1.43)	1.10 (0.84-1.45)	1.14 (0.88-1.48)	1.18 (0.93-1.51)
Grade III or IV IVH	10.0	1.13 (0.49-2.60)	1.31 (0.62-2.77)	1.51 (0.76-2.98)	1.74 (0.93-3.26)	2.01 (1.11-3.64)	2.32 (1.29-4.18)
	10.3	1.14 (0.53-2.44)	1.29 (0.65-2.57)	1.46 (0.78-2.73)	1.66 (0.93-2.94)	1.88 (1.09-3.23)	2.12 (1.25-3.60)
	10.8	1.16 (0.60-2.22)	1.27 (0.70-2.30)	1.39 (0.81-2.39)	1.52 (0.92-2.51)	1.67 (1.04-2.67)	1.83 (1.17-2.87)
	11.5	1.18 (0.69-2.02)	1.24 (0.76-2.03)	1.30 (0.82-2.05)	1.36 (0.88-2.08)	1.42 (0.94-2.14)	1.48 (1.00-2.21)
	12.2	1.21 (0.74-1.97)	1.21 (0.76-1.90)	1.21 (0.78-1.86)	1.21 (0.79-1.83)	1.21 (0.79-1.83)	1.21 (0.78-1.86)
	12.8	1.23 (0.73-2.08)	1.18 (0.73-1.91)	1.14 (0.72-1.80)	1.09 (0.69-1.73)	1.05 (0.65-1.70)	1.01 (0.60-1.70)
	13.1	1.24 (0.70-2.18)	1.17 (0.70-1.95)	1.10 (0.67-1.80)	1.04 (0.63-1.70)	0.98 (0.58-1.65)	0.92 (0.52-1.65)
In-hospital Mortality	10.0	1.47 (0.81-2.66)	1.59 (0.90-2.78)	1.71 (1.00-2.94)	1.85 (1.08-3.14)	1.99 (1.17-3.40)	2.15 (1.24-3.71)
	10.3	1.44 (0.84-2.46)	1.54 (0.93-2.57)	1.65 (1.01-2.69)	1.77 (1.10-2.85)	1.90 (1.19-3.04)	2.04 (1.27-3.28)
	10.8	1.39 (0.88-2.18)	1.47 (0.96-2.26)	1.56 (1.04-2.34)	1.66 (1.12-2.45)	1.76 (1.20-2.57)	1.86 (1.28-2.72)
	11.5	1.32 (0.93-1.89)	1.38 (0.9-1.92)	1.44 (1.05-1.98)	1.50 (1.10-2.05)	1.57 (1.15-2.14)	1.64 (1.19-2.26)
	12.2	1.26 (0.92-1.72)	1.29 (0.97-1.73)	1.33 (1.00-1.77)	1.37 (1.01-1.86)	1.41 (1.00-1.98)	1.44 (0.98-2.14)
	12.8	1.20 (0.86-1.69)	1.22 (0.90-1.67)	1.24 (0.90-1.71)	1.26 (0.87-1.82)	1.28 (0.83-1.98)	1.30 (0.77-2.18)
	13.1	1.18 (0.82-1.70)	1.19 (0.85-1.67)	1.20 (0.84-1.71)	1.21 (0.80-1.82)	1.22 (0.74-2.00)	1.23 (0.68-2.22)

Abbreviations: IVH, intraventricular hemorrhage; HR, hazard ratio; PCT, platelet count; MPV, mean platelet volume.

[a] For any grade of IVH and grade III or IV IVH outcomes, the Fine-Gray sub distribution hazard model was used to account for the competing risk of death. For in-hospital mortality outcome, Cox proportional hazards model was used. *P* values were calculated using corresponding models.

[b] Mean platelet volume of 10.0 µm³, 10.3 µm³, 10.8 µm³, 11.5 µm³, 12.2 µm³, 12.8 µm³, and 13.1 µm³ were selected beauce they are the 5th, 10th, 25th, 50th, 75th, 90th, and 95th percentile of the all mean platelet volume results of the study population.

[c] In all three models, the platelet count and mean platelet volume were included as time-varying variables and were defined as the most recent results at least one day prior to the outcome event. Two-way interaction terms between platelet count and platelet transfusion, between mean platelet volume and platelet transfusion, and three-way interaction term between platelet count, mean platelet volume, and platelet transfusion were included in all three models, so hazard ratio represented risk change for every additional platelet transfusion at certain platelet count and mean platelet volume levels. Despite of linear term, quadratic term of platelet count was added to the model of any grade of IVH, and quadratic and cubic terms were added to the model of in-hospital mortality since they were statistically significant. In all three models, infant sex, birthweight strata, gestational age strata, congenital disorder, singleton, any Apgar score ≤ 7 (1-min, 5-min, or 10-min Apgar), cesarean delivery, maternal preeclampsia, pregnancy-induced hypertension, antenatal steroids given, intrauterine distress, maternal High-risk pregnancy, and premature rupture of membrane were adjusted as time-constant covariates; first ventilation time after birth and the sepsis/necrotizing enterocolitis occurrence time after birth were adjusted as time-varying covariates.

© 2022 Chen C et al. *JAMA Network Open.*

eFigure 1. Association Between Platelet Transfusion–Associated Outcomes and Platelet Count, Extended

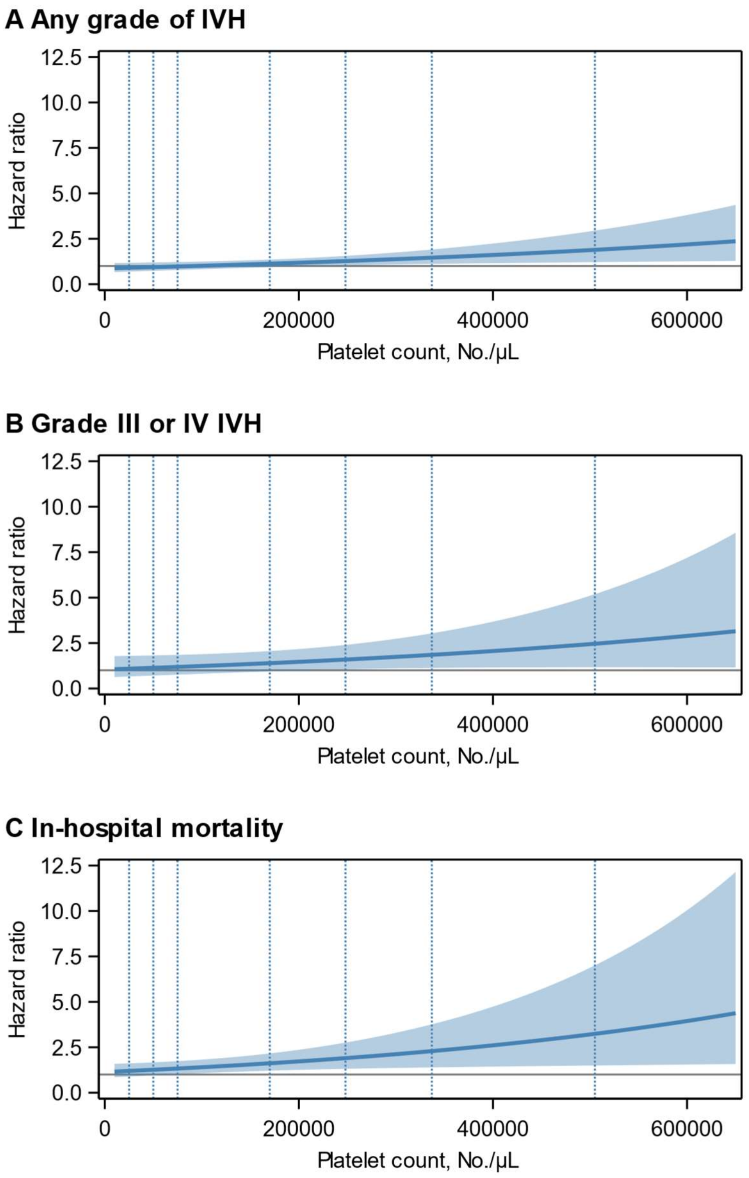

The dark line represents the hazard ratio, and the shaded area represents the 95% CI. Vertical dotted lines indicate platelet count values of 25 000 No./µL, 5 0000 No./µL, 75000 No./µL, 170000 No./µL, 248000 No./µL, 337000 No./µL, and 505000 No./µL. The last five values represented 5th, 25th, 50th, 75th, and 95th percentile of all platelet count results in the study sample. In panels A and B, hazard ratios were estimated by the Fine-Gray subdistribution hazard model to account for the competing risk of death. In panel C, the Cox proportional hazards model was used to estimate the hazard ratio. In three models, the platelet count was included as a time-varying variable, which was defined as its most recent result at least one day prior to the outcome event. The interaction term between platelet count and platelet transfusion was included in all three models, so the hazard ratio represents the risk change for every additional platelet transfusion at a certain platelet count level. Despite the linear term, the

© 2022 Chen C et al. *JAMA Network Open.*

quadratic term of platelet count was added to the model of any grade IVH, and quadratic and cubic terms were added to the model of in-hospital mortality since they were statistically significant. Additionally, in all three models, infant sex, birthweight strata, gestational age strata, congenital disorder, singleton, any Apgar score ≤ 7 (1-min, 5-min, or 10-min Apgar), cesarean delivery, maternal preeclampsia, pregnancy-induced hypertension, antenatal steroids given, intrauterine distress, maternal high-risk pregnancy, and premature rupture of membrane were adjusted as time-constant covariates; first ventilation time after birth and the sepsis/necrotizing enterocolitis occurrence time after birth were adjusted as time-varying covariates.

eFigure 2. Association Between Platelet Transfusion–Associated Outcomes and Platelet Distribution Width

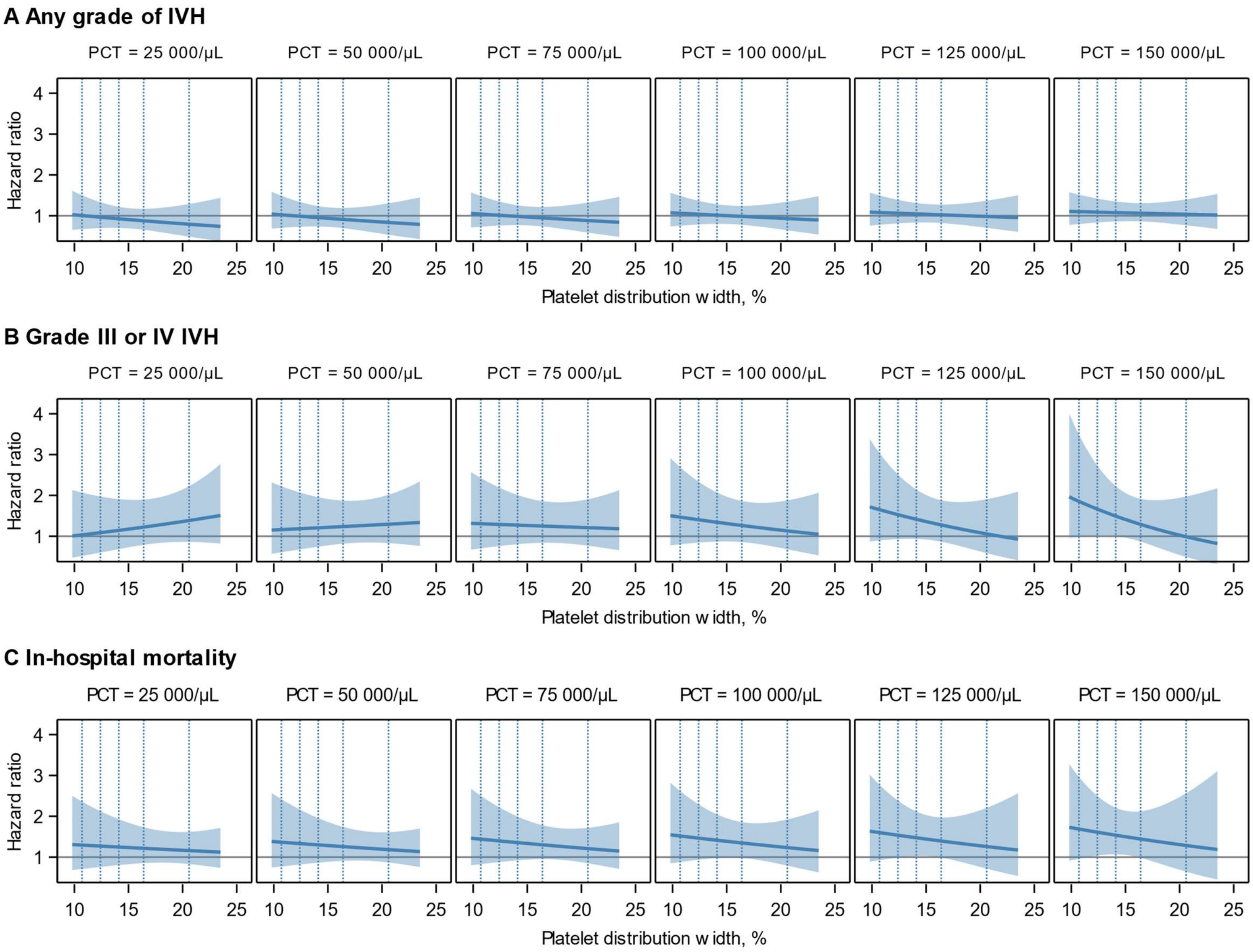

Abbreviation: PCT, platelet count.

To convert platelet distribution width to proportion of 1.0, multiply by 0.01.

© 2022 Chen C et al. *JAMA Network Open.*

The dark line represents the hazard ratio, and the shaded area represents the 95% CI. Vertical dotted lines indicate the 5th, 25th, 50th, 75th, and 95th percentiles of platelet distribution width records in the whole sample. In panels A and B, hazard ratios were estimated by the Fine-Gray subdistribution hazard model to account for the competing risk of death. In panel C, the Cox proportional hazards model was used to estimate the hazard ratio. In three models, the platelet count and platelet distribution width were included as time-varying variables, which were defined as the most recent test result at least one day prior to the outcome event. Additionally, infant sex, birthweight strata, gestational age strata, congenital disorder, singleton birth, Apgar score ≤ 7 (1-min, 5-min, or 10-min Apgar), cesarean delivery, maternal preeclampsia, pregnancy-induced hypertension, antenatal steroid use, intrauterine distress, maternal high-risk pregnancy, and premature rupture of membrane were adjusted as time-constant covariates. First ventilation time after birth and the sepsis/necrotizing enterocolitis occurrence time after birth were adjusted as time-varying covariates. In addition, two-way interaction terms between platelet count and platelet transfusion, between platelet distribution width and platelet transfusion, and three-way interaction terms between platelet count, platelet distribution width, and platelet transfusion were included in all three models. Despite the linear term, the quadratic term of platelet count was added to the model of any grade IVH, and quadratic and cubic terms were added to the model of in-hospital mortality.

© 2022 Chen C et al. *JAMA Network Open.*

eFigure 3. Association Between Platelet Transfusion-Associated Outcomes and Platelet–Large Cell Ratio

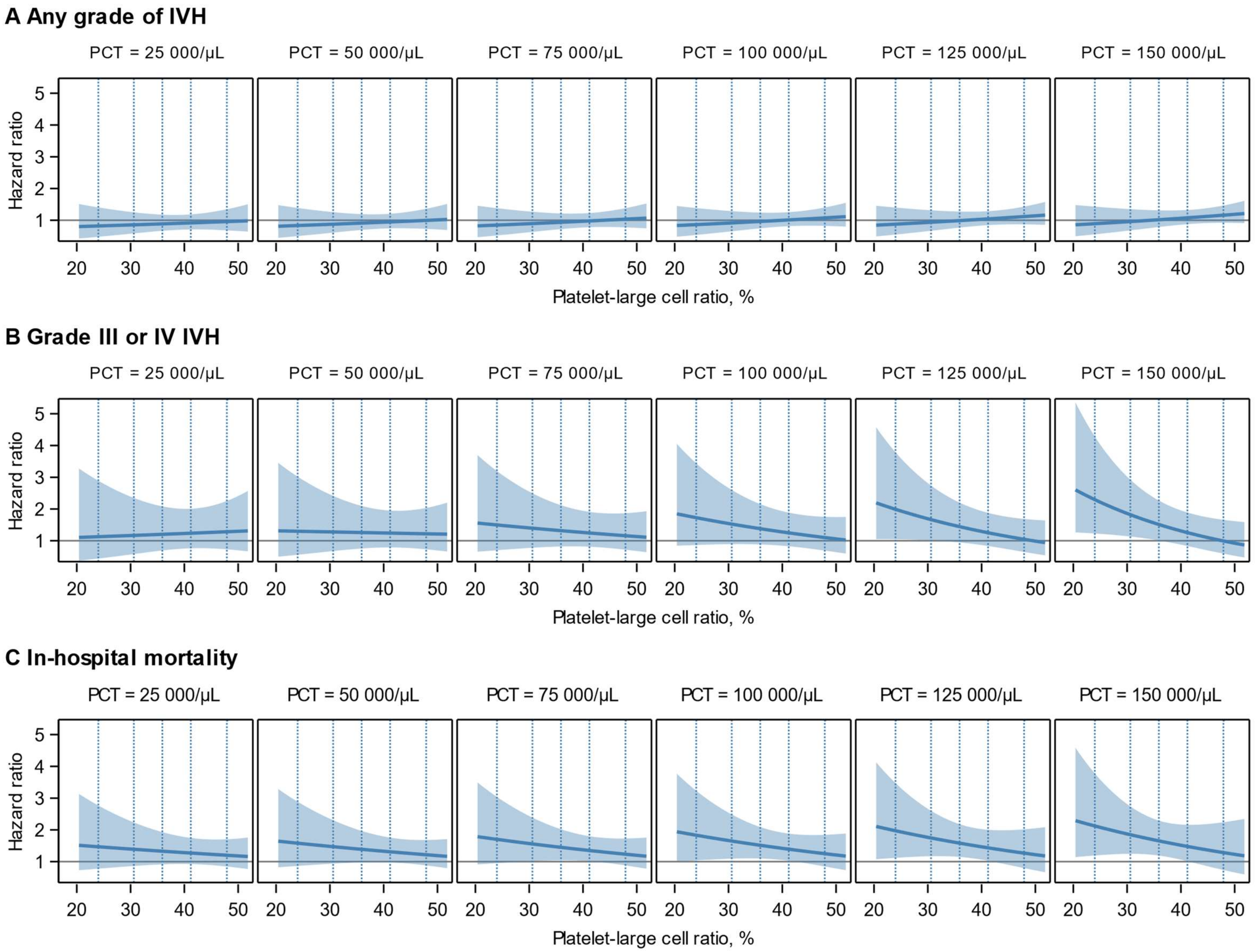

Abbreviation: PCT, platelet count.

To convert platelet-large cell ratio to proportion of 1.0, multiply by 0.01.

© 2022 Chen C et al. *JAMA Network Open.*

The dark line represents the hazard ratio, and the shaded area represents the 95% CI. Vertical dotted lines indicate the 5th, 25th, 50th, 75th, and 95th percentiles of platelet-large cell ratio records in the whole sample. In panels A and B, hazard ratios were estimated by the Fine-Gray subdistribution hazard model to account for the competing risk of death. In panel C, the Cox proportional hazards model was used to estimate the hazard ratio. In three models, the platelet count and platelet-large cell ratio were included as time-varying variables, which were defined as the most recent test result at least one day prior to the outcome event. Additionally, infant sex, birthweight strata, gestational age strata, congenital disorder, singleton birth, Apgar score ≤ 7 (1-min, 5-min, or 10-min Apgar), cesarean delivery, maternal preeclampsia, pregnancy-induced hypertension, antenatal steroid use, intrauterine distress, maternal high-risk pregnancy, and premature rupture of membrane were adjusted as time-constant covariates. First ventilation time after birth and the sepsis/necrotizing enterocolitis occurrence time after birth were adjusted as time-varying covariates. In addition, two-way interaction terms between platelet count and platelet transfusion, between platelet-large cell ratio and platelet transfusion, and three-way interaction terms between platelet count, platelet-large cell ratio, and platelet transfusion were included in all three models. Despite the linear term, the quadratic term of platelet count was added to the model of any grade IVH, and quadratic and cubic terms were added to the model of in-hospital mortality.

© 2022 Chen C et al. *JAMA Network Open.*

eFigure 4. Survival Plots Grouped by Pretransfusion Platelet Count at a Cutoff of 25 × 103/μL

Panel 1. For any grade IVH endpoint

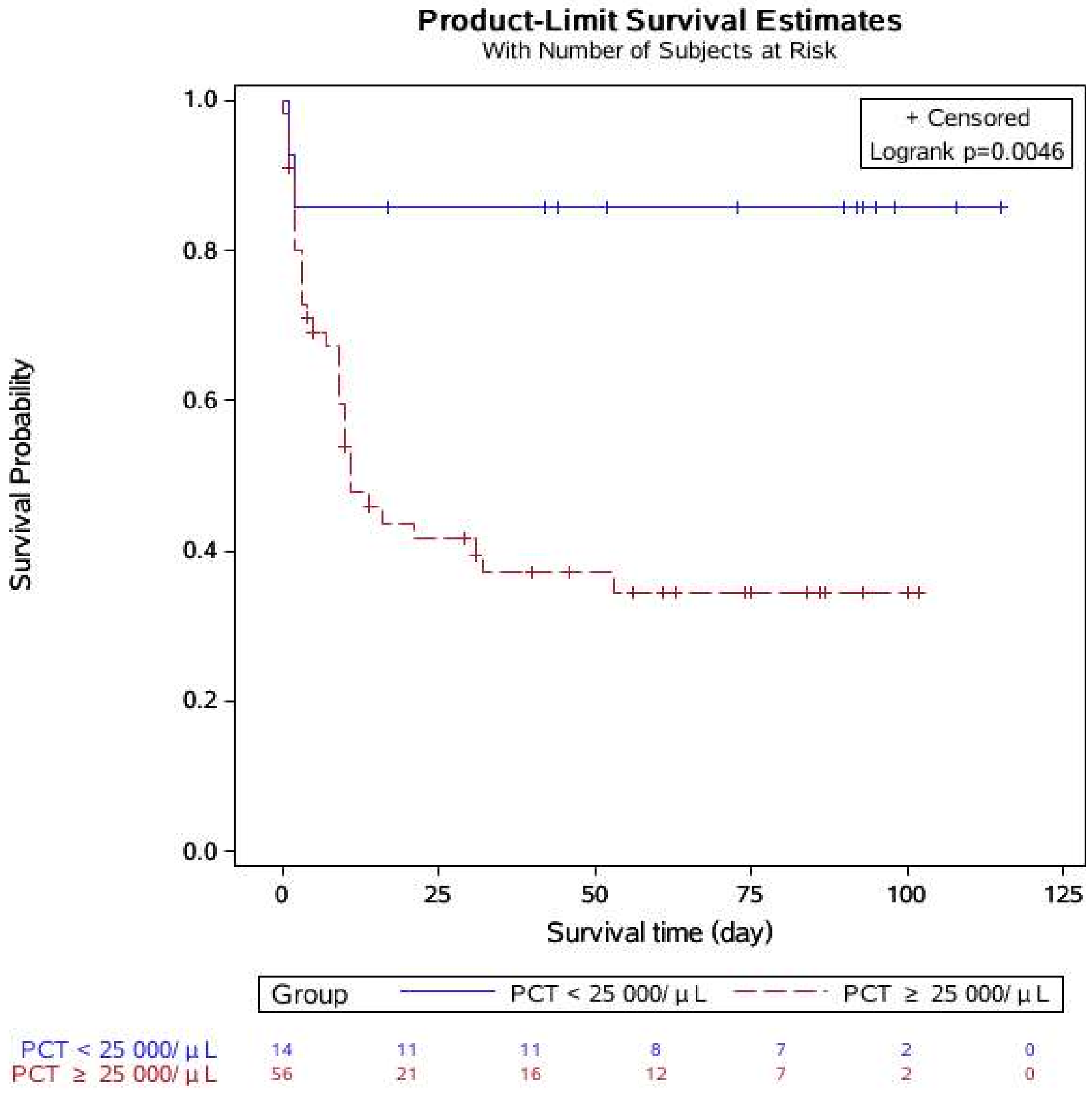

Abbreviation: PCT, platelet count.

Neonates who received prophylactic transfusions (a transfusion that occurred before any grade of IVH) with PCT records within the 24 hours (1 day) prior to the transfusion were selected to ensure the timeliness of the PCT. If multiple PCT records existed within the 24 hours prior to the transfusion, the lowest value was selected. Kaplan-Meier method was applied to estimate the survival curve. Log-rank test was performed.

© 2022 Chen C et al. *JAMA Network Open.*

Panel 2. For Grade III and IV IVH endpoint

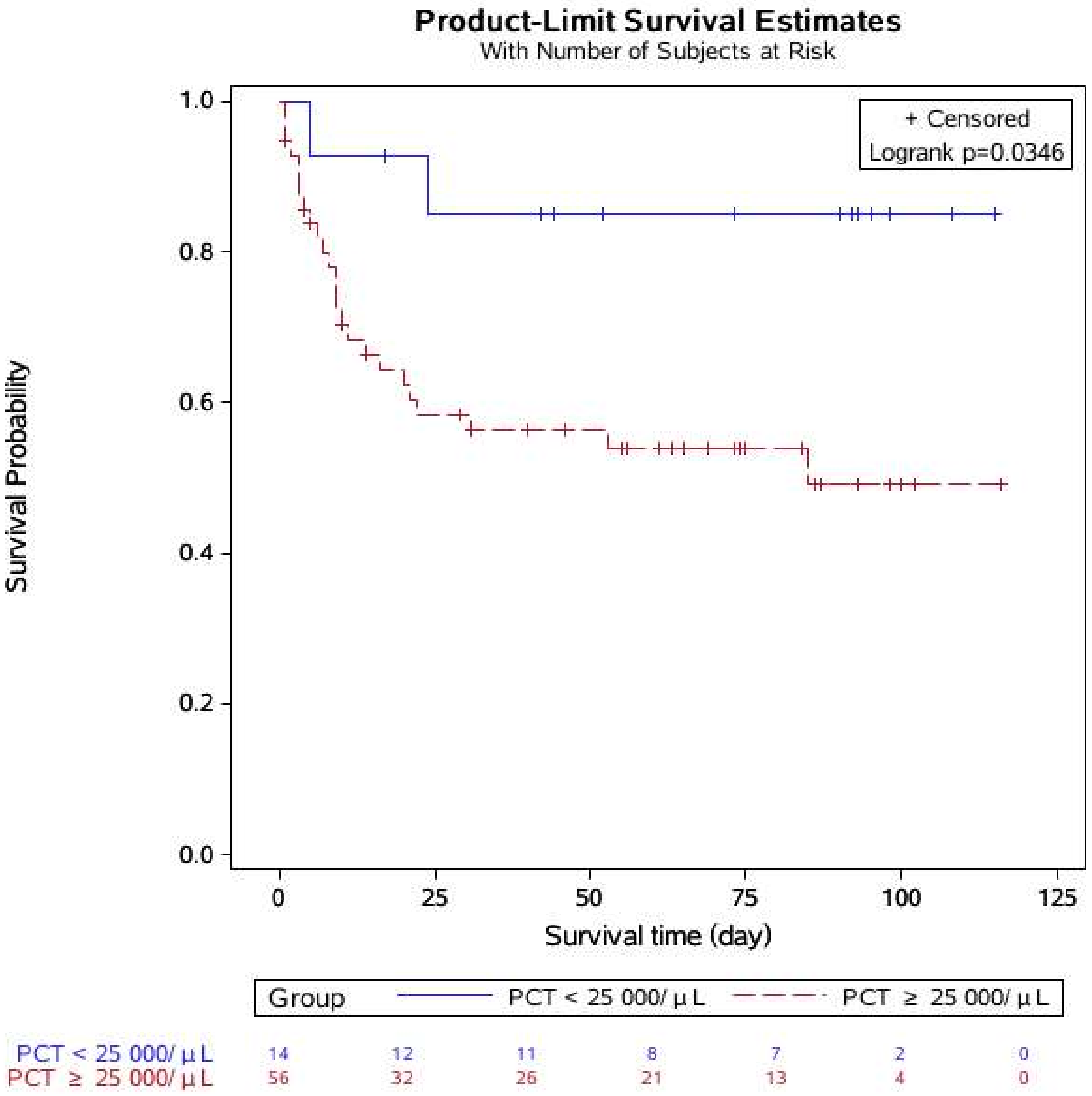

Abbreviation: PCT, platelet count.

Neonates who received prophylactic transfusions (a transfusion that occurred before any grade of IVH) with PCT records within the 24 hours (1 day) prior to the transfusion were selected to ensure the timeliness of the PCT. If multiple PCT records existed within the 24 hours prior to the transfusion, the lowest value was selected. Kaplan-Meier method was applied to estimate the survival curve. Log-rank test was performed.

© 2022 Chen C et al. *JAMA Network Open.*

Panel 3. For survival endpoint

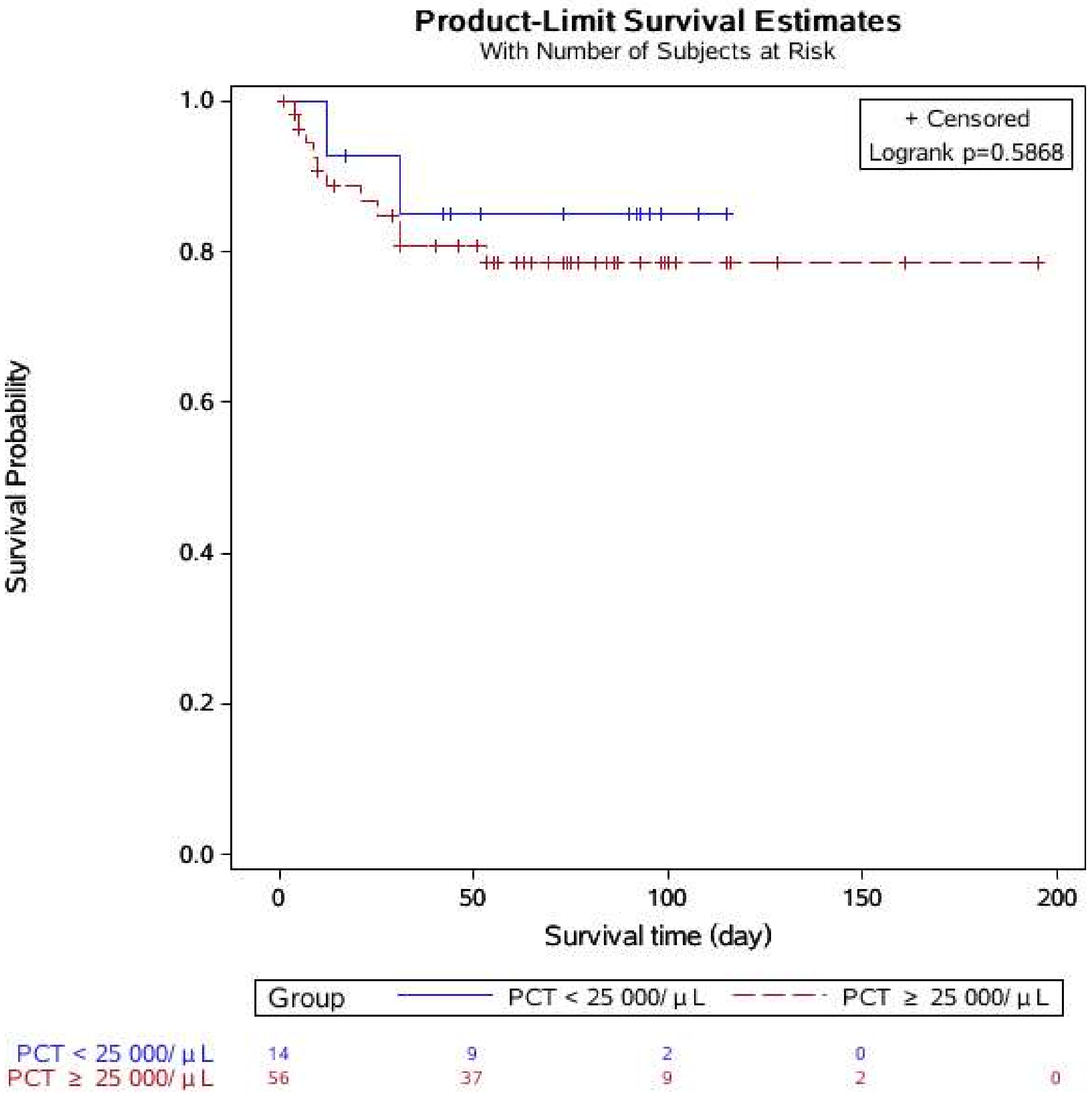

Abbreviation: PCT, platelet count.

Neonates who received prophylactic transfusions (a transfusion that occurred before any grade of IVH) with PCT records within the 24 hours (1 day) prior to the transfusion were selected to ensure the timeliness of the PCT. If multiple PCT records existed within the 24 hours prior to the transfusion, the lowest value was selected. Kaplan-Meier method was applied to estimate the survival curve. Log-rank test was performed.

© 2022 Chen C et al. *JAMA Network Open.*

eFigure 5. Survival Plots Grouped by Pretransfusion Platelet Count at a Cutoff of 50 × 103/µL

Panel 1. For any grade IVH endpoint

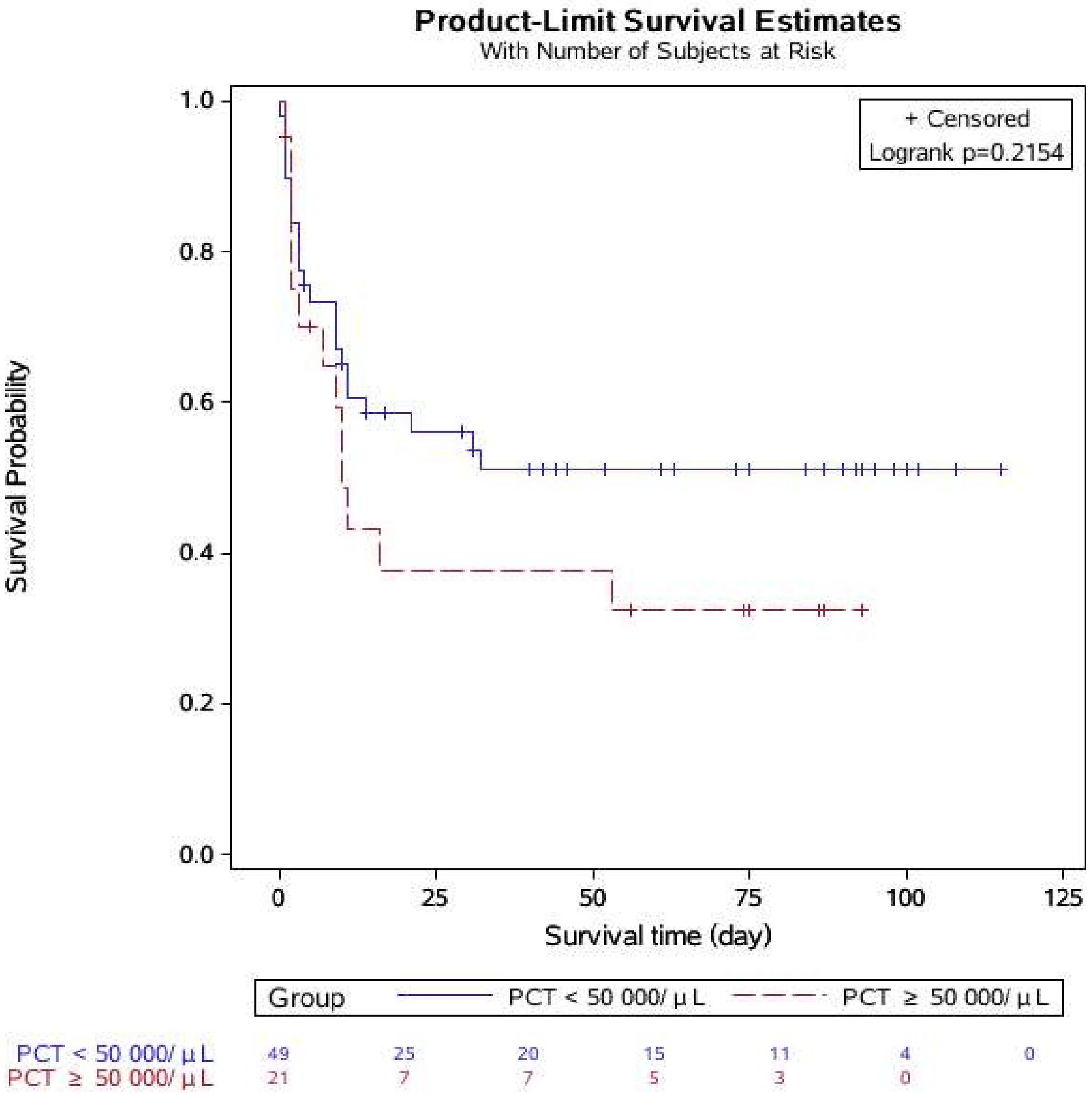

Abbreviation: PCT, platelet count.

Neonates who received prophylactic transfusions (a transfusion that occurred before any grade of IVH) with PCT records within the 24 hours (1 day) prior to the transfusion were selected to ensure the timeliness of the PCT. If multiple PCT records existed within the 24 hours prior to the transfusion, the lowest value was selected. Kaplan-Meier method was applied to estimate the survival curve. Log-rank test was performed.

© 2022 Chen C et al. *JAMA Network Open.*

Panel 2. For Grade III and IV IVH endpoint

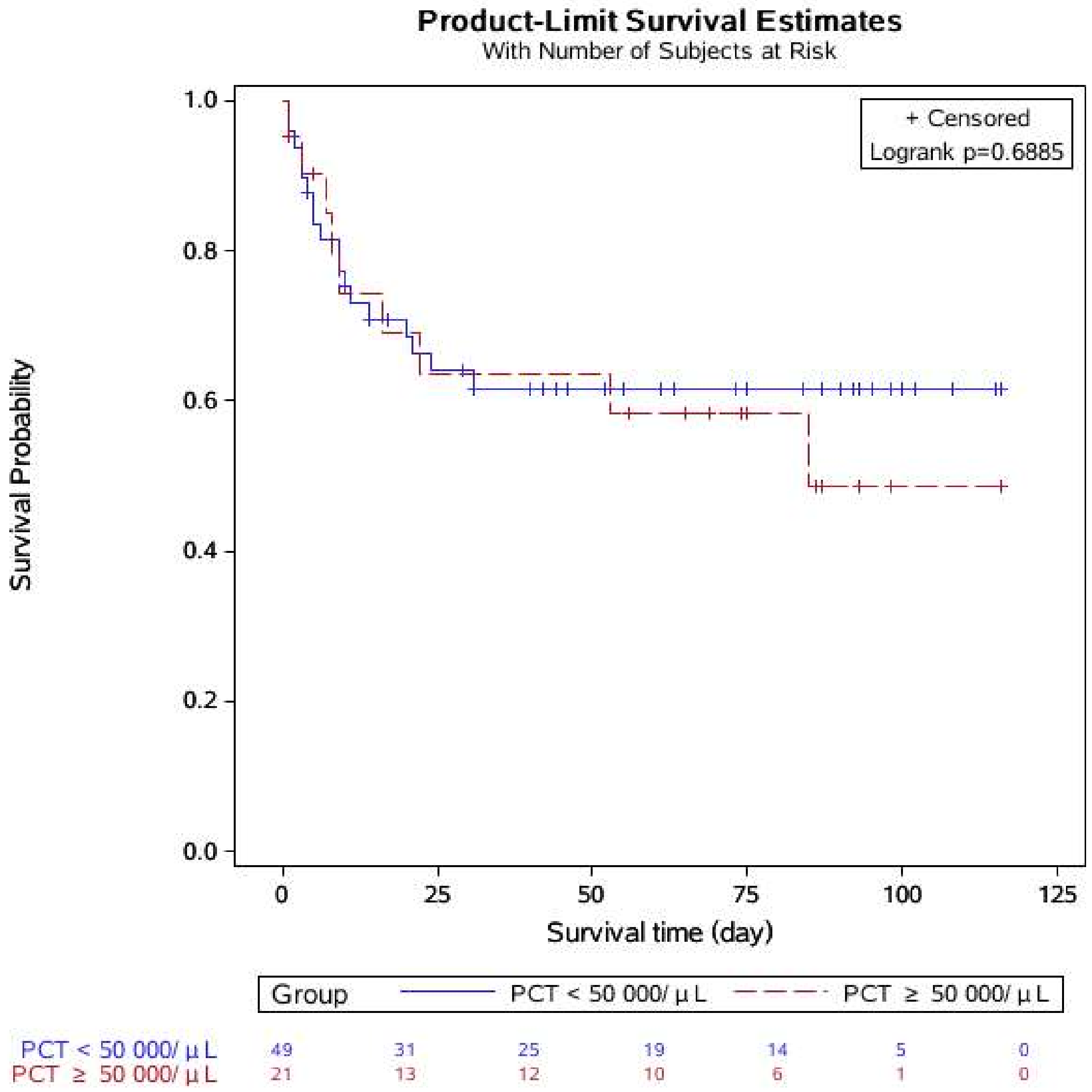

Abbreviation: PCT, platelet count.

Neonates who received prophylactic transfusions (a transfusion that occurred before any grade of IVH) with PCT records within the 24 hours (1 day) prior to the transfusion were selected to ensure the timeliness of the PCT. If multiple PCT records existed within the 24 hours prior to the transfusion, the lowest value was selected. Kaplan-Meier method was applied to estimate the survival curve. Log-rank test was performed.

© 2022 Chen C et al. *JAMA Network Open.*

Panel 3. For survival endpoint

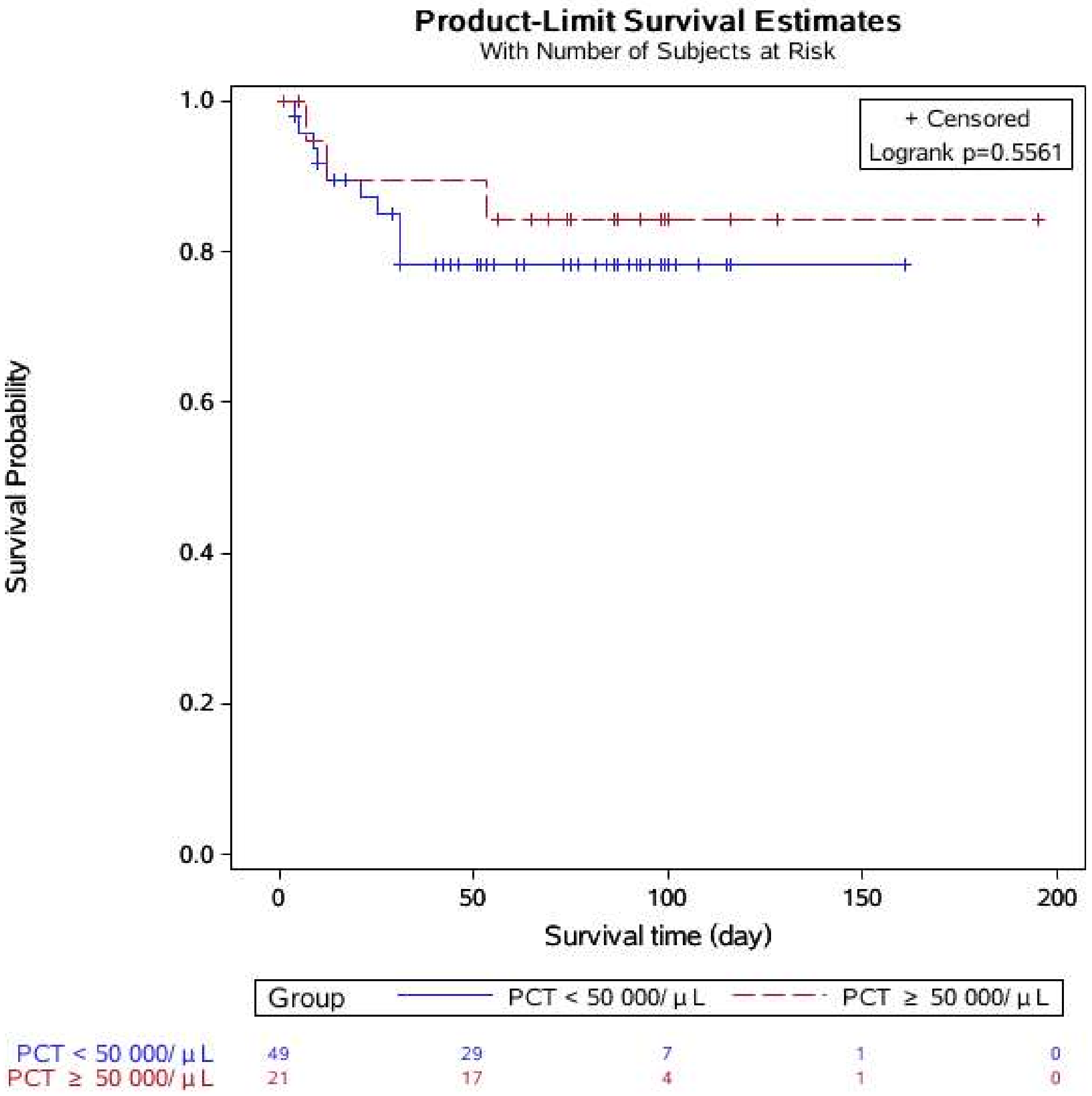

Abbreviation: PCT, platelet count.

Neonates who received prophylactic transfusions (a transfusion that occurred before any grade of IVH) with PCT records within the 24 hours (1 day) prior to the transfusion were selected to ensure the timeliness of the PCT. If multiple PCT records existed within the 24 hours prior to the transfusion, the lowest value was selected. Kaplan-Meier method was applied to estimate the survival curve. Log-rank test was performed.

© 2022 Chen C et al. *JAMA Network Open.*

eFigure 6. Flowchart of Selection of the Study Population

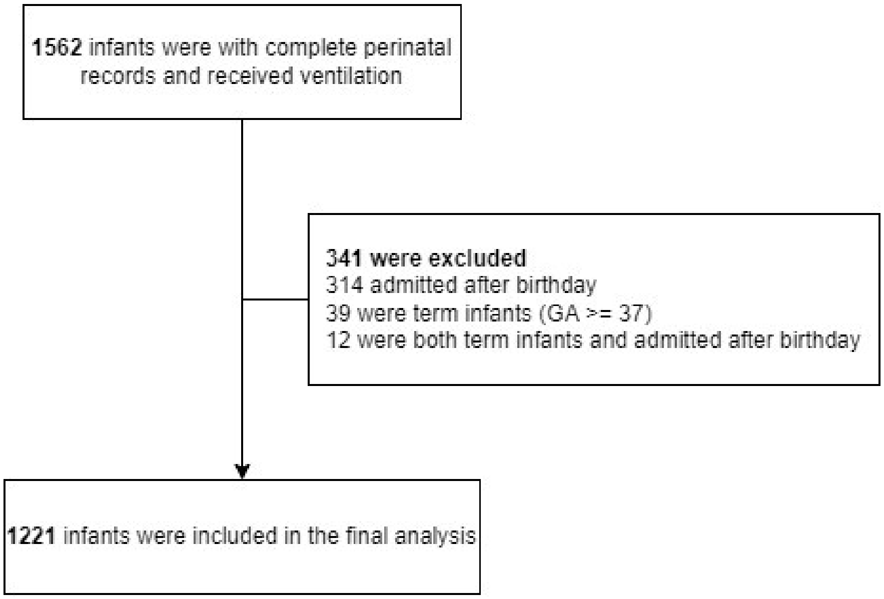

© 2022 Chen C et al. *JAMA Network Open.*

eMethods. Methods for Measuring Platelet Count and Mean Platelet Volumes and Reference Ranges

In our institution, platelet count and mean platelet volume are determined by an automated hematology analyzer (xs800i; Sysmex, Japan) according to the manufacturer's instructions. Briefly, blood cells wrapped by the sheath liquid pass one by one through a certain track within the detection region. Digital waveforms are originally recorded and transferred to physical parameters to depict the status of the platelet.

The normal reference range for the platelet count is 100 to 300×109/L, and the mean platelet volume is 7.6 to 15 fL.

© 2022 Chen C et al. *JAMA Network Open.*
